# Plasma Metabolite Profiling and Chemometric Analyses of Lung Cancer along with Three Controls through Gas Chromatography-Mass Spectrometry

**DOI:** 10.1038/srep08607

**Published:** 2015-02-25

**Authors:** Syed Ghulam Musharraf, Shumaila Mazhar, Muhammad Iqbal Choudhary, Nadeem Rizi, Atta-ur-Rahman

**Affiliations:** 1 Dr. Panjwani Center for Molecular Medicine and Drug Research, International Center for Chemical and Biological Sciences, University of Karachi, Karachi-. 75270, Pakistan; 2 H.E.J. Research Institute of Chemistry, International Center for Chemical and Biological Sciences, University of Karachi, Karachi-75270, Pakistan; 3 Department of Chemistry, College of Science, King Saud University, Riyadh-1145, Saudi Arabia; 4 Jinnah Postgraduate Medical Center, Karachi, Pakistan

## Abstract

Lung cancer has been the most common death causing cancer in the world for several decades. This study is focused on the metabolite profiling of plasma from lung cancer (LC) patients with three control groups including healthy non-smoker (NS), smokers (S) and chronic obstructive pulmonary disease patients (COPD) samples using gas chromatography-mass spectrometry (GC-MS) in order to identify the comparative and distinguishing metabolite pattern for lung cancer. Metabolites obtained were identified through National Institute of Standards and Technology (NIST) mass spectral (Wiley registry) and Fiehn Retention Time Lock (RTL) libraries. Mass Profiler Professional (MPP) Software was used for the alignment and for all the statistical analysis. 32 out of 1,877 aligned metabolites were significantly distinguished among three controls and lung cancer using *p*-value ≤ 0.001. Partial Least Square Discriminant Analysis (PLSDA) model was generated using statistically significant metabolites which on external validation provide high sensitivity (100%) and specificity (78.6%). Elevated level of fatty acids, glucose and acids were observed in lung cancer in comparison with control groups apparently due to enhanced glycolysis, gluconeogenesis, lipogenesis and acidosis, indicating the metabolic signature for lung cancer.

Lung cancer has been the most common death causing cancer in the world for several decades. Regardless of tremendous efforts, long-term survival has not improved significantly over the last 25 years. 5-Year survival rates of lung cancer patient remain only 15%^1^, which may increase up to 80%, if the lung cancer is detected in early stages^2^. According to the International Agency for Research on Cancer (IARC) for 2012 report, one of the most frequent cancers in the world is lung cancer which has the highest incidence rate worldwide (1.8 million, 13% of the total). As far as the mortality rate is concern, lung cancer is again at the top (1.6 million, 19.4% of the total)^3^. Several studies have been conducted on molecular biomarkers for the early detection of lung cancer at genomics, epigenomics, proteomics, and metabolomics levels^4^,^5^,^6^,^7^ to reduce their mortality rate. Metabolomics in the post-genomic era is a powerful tool for profiling differences in metabolites among normal, precancerous, and cancerous cells or tissues. Moreover, metabolomics has gained considerable importance due to recent advances in experimental methodologies and technologies, and ability to process large amounts of data. Based on this, metabolomics approaches can permit early diagnosis or real-time monitoring of the effects of a disease^8^.

The metabolic studies of lung cancer in human tissues and biofluids have been reported in the last few years. Kenjiro Kami *et al*., have reported metabolomic profiling of lung and prostate tumor tissues by Capillary Electrophoresis Mass Spectrometry (CE-MS)^9^. Rocha *et al*., have studied the metabolic differentiation between tumor and non-involved adjacent lung tissues by High Resolution Magical Angle Spinning Nuclear Magnetic Resonance (HRMAS-NMR) spectroscopy^10^. They investigated increased levels of lactate, phosphocholine (PC), and glycerophosphocholine (GPC) in tumors, while glucose, myo-inositol, inosine/adenosine and acetate level were decreased. Carrola *et al*, investigated the Nuclear Magnetic Resonance (NMR) based metabonomics in blood plasma and urine^11^ for metabolic signatures in lung cancer. Using a more global profiling approach, Jordan and colleagues reported the NMR analysis of paired tissues and serum samples from 14 subjects with two different lung cancer histological types (adenocarcinoma and squamous cell carcinoma), as well as of serum from 7 healthy individuals^12^. In another pubilcation, a panel of 8 metabolites were identified for the diagnosis of breast, lung, colon or prostate cancers with a high sensitivity and specificity^13^.

A few targeted metabolic profiling of blood plasma/serum have been reported for lung cancer biomarkers discovery. Maeda and co-workers reported the differences in the amino acid profiling of plasma between healthy controls and non-small-cell lung cancer (NSCLC) patients, as assessed by Liquid Chromatography Mass Spectrometry (LC/MS)^14^. Targeted analysis of lysophosphatidylcholines (lysoPC) showed that irregular levels of lysoPC isomers with different fatty acyl positions were found in the plasma of lung cancer patients as compared to controls^15^. In another targeted analysis, serum lipid metabolite profiling of 58 lung cancer using Fourier transform ion cyclotron resonance MS has been reported^16^.

Recent advances in NMR, GC-MS and LC/MS techniques have enabled the use of more global metabolomic approaches for the identification of novel biomarkers for specific diseases^7^,^17^,^18^ as well as new targets for drug discovery and development. Among the recent techniques, GC-MS proved to be a significantly useful method due to its high sensitivity and resolution, reproducibility and cost effectiveness. Moreover, in comparison to LC/MS, the availability of a large GC-MS electron impact (EI) spectral library further aids the identification of biomarkers in various pathological condition^19^. There are few reports published based on GC-MS analysis of lung cancer metabolites. Metabolites in serum and urine of 19 lung cancer patients and 15 patients with other lung diseases were analyzed using GC-MS^20^. Serum metabolomic analysis of lung cancer patients was performed using GC-MS from 29 healthy volunteers and 33 lung cancer patients^7^. Few studies on GC-MS based volatile organic compounds (VOC) as lung cancer biomarkers have also been reported^21^,^22^,^23^,^24^,^25^.

In all above cited investigations, either limited numbers of samples were used or one healthy control group was used to discriminate lung cancer metabolites. In the present study, we have used 384 samples with three control groups including healthy non-smokers, smokers and persons with COPD in order to identify diseases related metabolites through comprehensive comparison. Previously, we have developed a comprehensive, straightforward, reproducible and efficient sample preparation method which can cover a wide range of metabolites for metabolite profiling with 2D-C18 fractionation approach^26^. In this investigation, all the samples were analyzed through 2D-C18 method for the first time to investigate differentiative metabolite patterns between the lung cancer and controls, followed by chemometric analyses.

## Methods

### Solvents and reagents

All solvents used for GC-MS analysis were of analytical grade. Methanol, hexane and ammonium hydroxide were purchased from Tedia (Tedia way, Fairfield, USA), while isopropanol and hydrochloric acid (37%) were purchased from Fisher Scientific (Loughborough, Leicestershire, U.K.), formic acid and myristic-d_27_ acid were purchased from Sigma-Aldrich (St. Louis, MO, USA, respectively). MSTFA (*N*-Methyl-*N*- (trimethylsilyl) trifluoroacetamide) and methoxylamine hydrochloric were purchased from Acros Organic (New Jersey, USA). Deionized water (Milli-Q) was used throughout the study (Millipore, Billerica, MA, USA).

### Sample collection statistics of patients and controls

This study was approved by the ethical committee of the Jinnah Postgraduate Medical Center (JPMC), and written informed consent was obtained from all the participants. A total of 384 plasma samples of healthy Non-Smokers (NS), Smokers (S), and Chronic Obstructive Pulmonary Disease (COPD) and Lung Cancer (LC) patients were included in this study. 96 samples from each group in the age range of 30–65 years among S and NS, while 35–70 years in the case of COPD and LC patients were selected. Cancer subjects included in this study were of pathologically proven LC of common subtypes, including 10 Squamous Cell Lung Cancer (SqLC), 12 Adenocarcinoma Lung Cancer (AdLC), 16 Small Cell Lung Cancer (SmLC), 10 Non Small Cell Lung Cancer (NSCLC) and 52 were uncategorized Lung Cancer (type of lung cancer were not diagnosed). The smokers included in this study had been smoking for at least 10 years or more.

Blood samples of male and female were collected from the JPMC Karachi, Pakistan, after consent. About 8 mL of the blood was drawn in the morning from the overnight fasting volunteers in BD Vacutainer tubes (BD Franklin Lakes, NJ, USA, REF 367856), containing K_2_-ethylenediaminetetraacetic acid as an anticoagulant. Plasma was separated immediately by centrifugation at 4,500 rpm for 10 min at 4°C. Finally, the plasma was aliquoted and frozen at −80°C. A code was given to each sample. Sample collection description and codes are mentioned in Table 1&2.

### Sample preparation

Method was carried out in accordance with our previous protocol^26^ with some modification. Samples were processed in a 96-well plate, in each plate aliquots of 100 μL of plasma of each samples were mixed with 800 μL of solvent methanol, 20 μL of internal standard myristic-d_27_ acid (1 mg/mL stock solution) was added and left on ice for 30 minutes. The precipitated proteins were then removed by centrifugation at 12,000 rpm for 10 min (Eppendorf Centrifuge 5804 C/R). Aliquots (600 μL) of the resulting clear supernatants were loaded onto the C18 96-well plate (Strata C18-E, 55 μm pore size, 70°A particle, 100 mg sorbent/well Phenomenex, USA) and drawn through the solid phase under vacuum. Prior to extraction, the phase was activated with 2 × 300 μL of methanol and then further conditioned with 2 × 300 μL of water. After loading of sample on plate, the phase was washed with 2 × 200 μL of water and eluted with 600 μL of methanol. The eluates were collected in 96-well collection plates. The eluate was then evaporated under N_2_ at room temperature. The dry samples were stored at 4°C until analysis. The SPE extractions were performed on solid phase extraction vacuum manifold AH0-7502 Phenomenex (USA).

### Derivatization and GC-MS analysis

The dried extract of all the samples were derivatized subsequently by adding 50 μL methoxylamine hydrochloride in pyridine (15 μg/μL), vortexed and left for 2 hr at 35°C. Then BSTFA was added with 1% trimethylchlorosilane (TCMS) and placed at 70°Cfor 60 min to form trimethylsilyl (TMS) derivatives. GC-MS parameters were same as those reported in our previous paper^26^. GC-MS analysis was performed using 7890A gas chromatography (Agilent technologies, USA), equipped with an Agilent Technology GC sampler 120 (PAL LHX-AG12) autosampler and coupled to a Agilent 7000 Triple Quad system (Agilent technologies, USA) and HP-5MS 30 m–250 mm (i.d.) fused-silica capillary column (Agilent J&W Scientific, Folsom, CA, USA), chemically bonded with a 5% diphenyl 95% dimethylpolysiloxane cross-linked stationary phase (0.25 mm film thickness) according to our previous report^26^.

### GC-MS data preprocessing and statistical analysis

Metabolite profiling of blood samples were analyzed using the optimized GC-MS assay. Data processing was performed using the Agilent Mass Hunter Qualitative Analysis (version B.04.00). Peak integration and deconvolution (parameter were same as previously reported except SNR threshold 3.0^26^ were performed on Mass Hunter. Putative identification of low molecular weight metabolites were established by comparing the mass spectra of the peaks with those available in the NIST mass spectral (Wiley registry NIST 11) and Fiehn RTL libraries. The identification of peaks was based on 70% similarity index. All the GC-MS spectra were exported as CEF format, and uploaded on MPP for peak alignment, normalization, significance testing, fold change and multivariate analysis for both identified and unidentified compounds.

All the available data (full scan mode from *m/z* 50 to 650 and retention time window 6.5 to 35 min) and minimum absolute abundance of 5,000 counts were used to filter the data. Alignment parameter was set as retention time tolerance 0.05, match factor 0.3 and delta MZ 0.2. Data was normalized to unit scale. After the normalization of data, baseline differences in metabolism between the four groups were eliminated. For baseline correction, all the compounds treated equally regardless of their intensity. It subtracts the mean abundance of each entity from the corresponding values in each sample. A total of 1,877 entities were found in the entire samples after alignment. Entities were filtered by frequency (those which appeared in more than 50% of samples in at least one group of samples were chosen), *p* ≤ 0.001, fold change> 3 and coefficient of variance (CV) < 25%. Statistical significance analysis using the one way ANOVA and a level of probability of 0.001 was used as the criterion for significance. 32 Entities were found to be significantly different in lung cancer and controls. Turkey's honest Significance Difference (HSD) post Hoc test was then applied to identify which entities were responsible for significant differences in the four groups. Hierarchical clustering was performed by applying Pearson's uncentered-absolute distance metric, complete linkage. Class prediction was built using a PLSDA model. PLSDA was constructed using 32 entities of filtered data using four components including auto scaling, N fold validation type, three numbers of fold and with ten numbers of repeats. Sensitivity and specificity were also measured from the construct model. 40 Samples were randomly selected and validated through the constructed model.

## Results and discussion

Metabolite profiling of a total 384 plasma samples from healthy non-smokers, smokers, COPD and lung cancer patients (96 samples of each group) were analyzed by using GC-MS. 2D-C18 sample preparation method was used for the enrichment of metabolites based on our previous findings^26^. Data files were subjected to extensive statistical analysis using MPP software in order to identify the comparative and statistically distinguished metabolites for the search of lung cancer biomarkers.

### Significance testing and fold change

The purpose of significant testing and fold change is to identify statistically differentiative metabolites by applying appropriate test and conditions. Thirty two metabolites, out of 1,877 were found to be significantly different among the three controls (NS, S and COPD) and lung cancer using one way ANOVA and a level of probability of 0.001 and fold change > 3 (Table 3). Eleven metabolites i.e. lactic acid (CAS # 79-33-4), phosphoric acid (CAS # 7664-38-2), benzoic acid (CAS # 2078-12-8), naphthalene (CAS # 29422-13-7), d-glucose (CAS # 128705-73-7), altrose (CAS # 1990-29-0), palmitic acid (CAS # 64519-82-0), octadecanoic acid (CAS # 1188-75-6), stearic acid (CAS # 57-11-4), 1-propene (CAS # 1000154-23-3) and cholesterol (CAS # 1856-05-9), out of 32 low molecular weight metabolites were putatively identified (level 2 of Metabolomics Standard Initiative for the identification) by comparing the mass spectra of the peaks with those available in the NIST mass spectral (Wiley registry NIST 11) and Fiehn RTL libraries at 70% similarity index (Table 3), while the remaining were not identified at this similarity index (Table 3). The EI/MS spectra of unidentified compounds are shown in supplementary information (Fig. S1).

**Figure 1:**
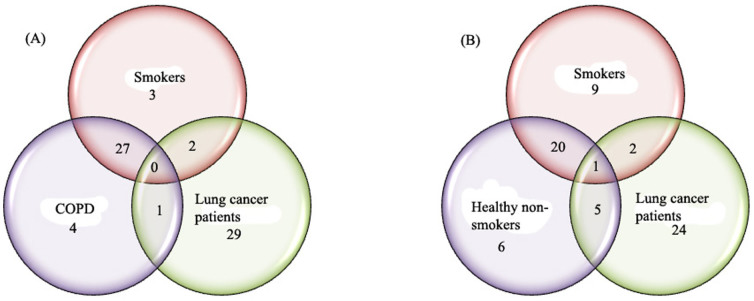
Venn diagrams highlighting the overlapping of statistically differentiative metabolites observed (A) among smoker, COPD and lung cancer patients, (B) among healthy non-smokers, smokers and lung cancer patients samples by applying Turkey's honest significance difference HSD post Hoc test.

After the completion of ANOVA, Turkey's honest significant difference (HSD) post Hoc test was applied in order to find out which entities or metabolites were significantly expressed among controls and lung cancer. It was found that a large number of metabolites were significantly different in lung cancer and the three control groups. 31 in COPD, 30 in smoker and 27 metabolites in healthy were significantly expressed, as compared to lung cancer. Only five metabolites were statistically different in smoker and COPD, showing the close resemblance between these two groups. 11 and 12 metabolites in healthy groups were statistically significant, as compared to COPD and smoker, respectively. Turkey's honest significant difference (HSD) post Hoc test summary is shown in supplementary information (Table S1) while identities of statistically significant metabolites which were differing in the four groups are also provided in supplementary information (Table S2). Venn diagram shows the overlapping of statistically differentiative metabolites between controls and lung cancer. In comparison of lung cancer with smoker and COPD, no peaks were overlapped in all the samples. 27 out of 32 were overlapped in smokers and COPD showing their close resemblance. However, 29 peaks were unique in lung cancer group which created differences between lung cancer and controls, while only 1 and 2 peaks were overlapped between lung cancer with COPD and smokers, respectively (Fig. 1A). In contrast, comparison of lung cancer with smokers and healthy non-smokers showed only 1 overlap peak in all samples, while 20 peaks were overlapped in healthy non-smokers and smokers. In this comparison, 24 peaks were unique to lung cancer which created a difference between lung cancer and controls, while only 2 and 5 peaks were overlapped between the lung cancer with smokers and healthy non-smokers, respectively (Fig. 1B).

### Clustering

**Figure 2:**
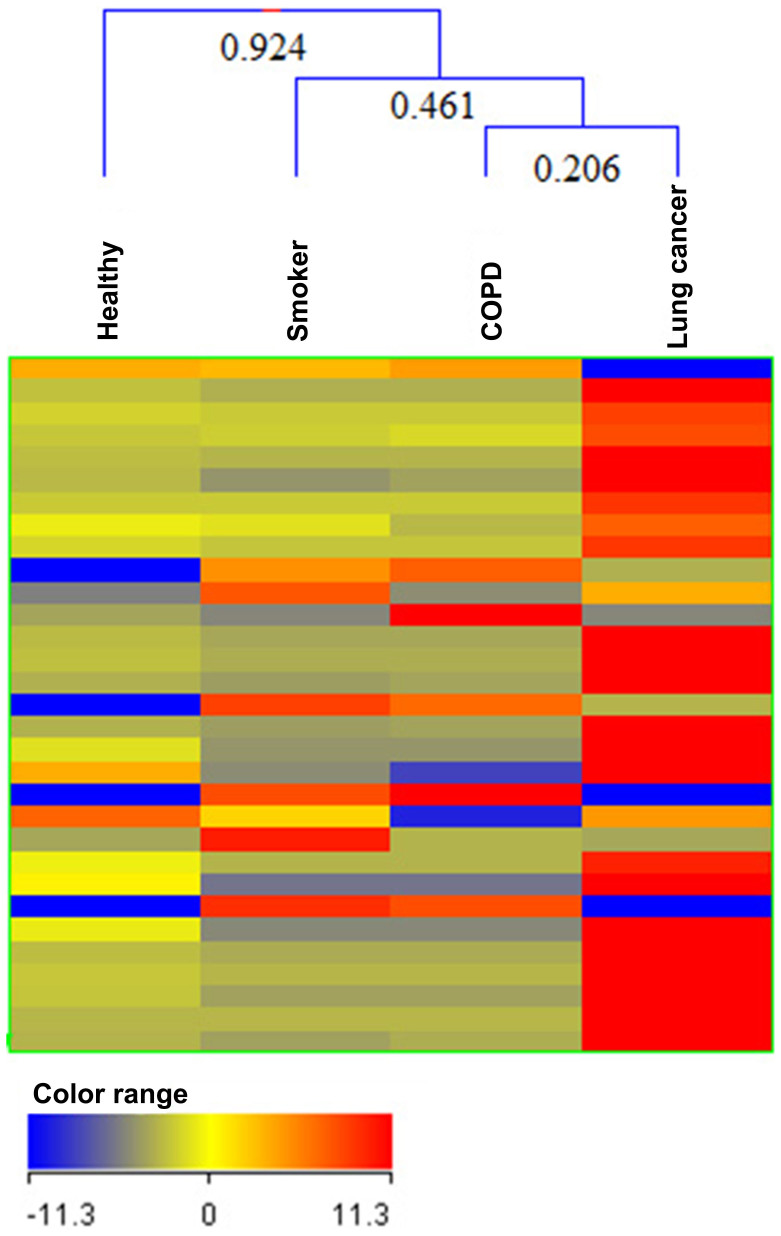
Comparison of four groups of samples i.e. healthy non-smokers (NS), smokers (S), Chronic Obstructive Pulmonary Disease (COPD) and Lung Cancer (LC) patients using normalized intensities of thirty two significance metabolites. The dendrogram was produced by applying a hierarchical clustering algorithm (Pearson's uncentered-absolute distance metric, Complete Linkage).

**Figure 3:**
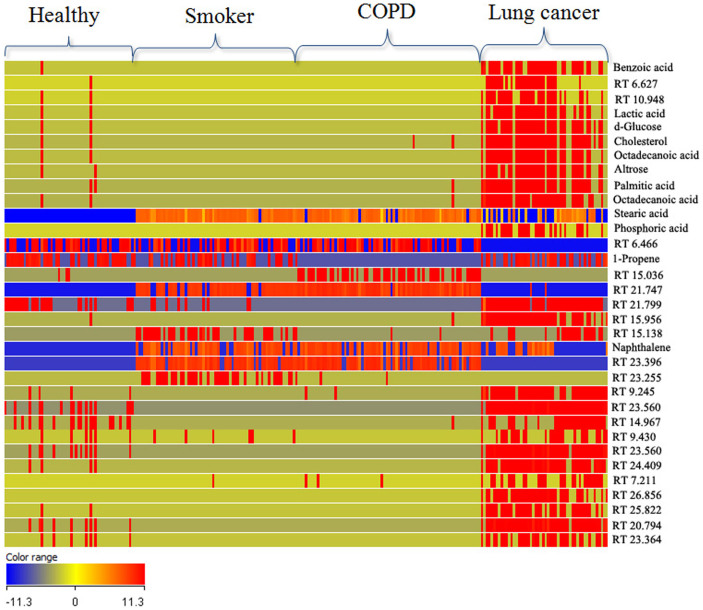
Heat map of all analyzed samples with normalized intensities of thirty two statistically significance metabolites. Identified compounds are labeled by their name while unidentified compounds are labeled by their retention time (RT).

Cluster analysis is a powerful method to organize either entities (compounds) or groups of samples into clusters, based on the similarity of their profiles. Hierarchical clustering was performed to produce a dendrogram for clustering of samples groups using normalized intensities of thirty two significance metabolites (Fig. 2). The length of the vertical lines in the dendrogram is a measure of dissimilarity, while shorter lines demonstrate close relationship of the groups. This approach clustered the four groups (three controls and lung cancer group) into classes I, II and III (Fig. 2). The two groups, i.e. lung cancer (LC) and COPD clustered together in class I with dissimilarity level of only 0.206 (Fig. 2). In class II, three groups, i.e. LC, COPD and smokers (S) were at dissimilarity level of 0.461 (Fig. 2). Clustering of all the four groups in class III showing dissimilarity level of 0.924 (Fig. 2) indicated that healthy non-smokers (NS) are most dissimilar from among the three groups, i.e. S, COPD and LC. Almost all the LC and COPD patients possess smoking background which results in close relationship of the three groups. An image of heat map using non-average samples (visualizing all samples) with normalized intensities of thirty two significant metabolites is shown in Fig. 3. From this figure, it is clear that lung cancer profile is totally different from three controls by considering all the samples of each group. There is also good reproducibility in each group and mostly the significantly differentiative metabolites are highly expressed in lung cancer as compare to control ones. Each histological subgroup of lung cancer was also compared with control groups (Fig. S3 of supplementary material). Squamous cell carcinoma and small cell carcinoma of lung cancer are strongly related with smoking habit and this is also supporting in our clustering analysis of significance metabolites in Fig. S3(A and B) while adenocarcinoma of lung cancer were not clustered with smokers, as adenocarcinoma is the most common form of lung cancer among people who have often or never smoked in their lifetimes Fig. S3C. Non-small cell lung cancer were also not clustered with smokers, this may be due to most of the samples in this class have adenocarcinoma (a type of non-small cell) Fig. S3D.

### Class prediction model and test

**Figure 4:**
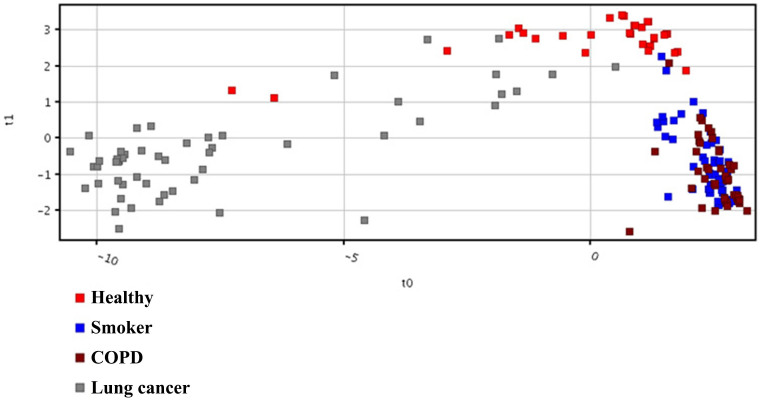
PLS-DA Scores scatter plots discriminating among controls and lung cancer patients based on the thirty two significantly differentiate metabolite profiling data. The red, blue, brown and gray squares indicate healthy volunteers (n = 54), smokers (n = 66), COPD (n = 75) and lung cancer patients (n = 52), respectively.

A model was built using thirty two statistically significant metabolites. Partial Least Square Discrimination (PLSD) algorithm was used to classify samples into discrete classes. The classes in the input data are randomly divided into three equal parts; two parts were used for training, and the remaining part was used for testing. The process was repeated ten times with a different part that is used for testing in each iteration. Thus each row is used at least once in training and testing, and a Confusion Matrix is generated. The results of Confusion Matrix (a matrix which gives the accuracy of prediction of each class) are presented in supplementary information Table S3. Figure 4 shows the plots obtained by PLS-DA scores. A clear separation trend was observed between the three controls involving healthy non-smokers, smokers and COPD with lung cancer samples in the PLS-DA scores plot (Fig. 4). The smokers and COPD lies close to each other as there are 27 entities were common between them (Fig. 1A). The lung cancer group was totally different from the controls groups as there were at least 24 entities significantly different from the controls in the lung cancer group (Figure 1) and this is also seen in the heat map (Fig. 3). Sensitivity and specificity are also measured from the constructed model. Sensitivity was calculated from the ratio of true positives (cancer samples which correctly predicted) to the total number of subjected cancer samples, whereas specificity was determined from the ratio of true negatives (control samples which correctly predicted) to the total number of subjected control samples. Sensitivity and specificity was found to be 96.2% and 92.0%, respectively, and overall accuracy of the model was found to be 93.1%. External validation measures the predictive capability (sensitivity and specificity) of a calculated model. The model was used to externally validate an independent or blind-test set of 38 plasma samples (8 healthy non-smokers, 10 smokers, 10 COPD and 10 lung cancer patients). PLSDA classifier correctly predicted the presence of LC in 10 out of 10 patients, healthy non-smokers in 8 out of 8, COPD 9 out of 10 and smokers 5 out of 10 resulting with 100% sensitivity and 78.6% specificity. 50% of the smokers were incorrectly predicted by the model as COPD, may be due to the common smoking history of both. All the sample prediction reports are shown in Figure S2 of supporting information.

### Pathway analysis

Pathway analysis was done through MPP software using thirty two significantly differentiative metabolites which reveals disturbance in several pathways including pyruvate metabolism and citric acid (TCA) cycle, fatty acid triacylglycerol and ketone body metabolism, bile acid and bile salt metabolism, ATP Binding Cassette (ABC) family protein mediated transport and G-Protein Coupled Receptor (GPCR) downstream signaling pathways.

### Pyruvate metabolism and citric acid (tca) cycle

All cells in our bodies require oxygen and nutrients. Energy is constantly needed to perform cellular functions. For the proliferation of cells, nutrients are needed in abundance for rapid growth. Therefore, cancer cells require a plentiful supply of nutrients. Most cancer cells are highly dependent on glucose for energy. Our experimental data showed that the level of glucose was different between lung cancer and control plasma samples. High levels of glucose were found in the plasma samples of lung cancer, as compared to controls. Warburg reported the conversion of glucose to lactic acid in the presence of oxygen as a specific metabolic abnormality of cancer cells^27^(Mishra and Verma, 2010). High level of lactic acid was also found in the plasma samples of lung cancer. High level of glucose in lung cancer does not show the decrease in glycolysis as lactic acid is also up-regulated in lung cancer. Glycolysis results in the breakdown of glucose, but several reactions in the glycolysis pathway are reversible and participate in the re-synthesis of glucose, so gluconeogenesis may be responsible for the increased levels of glucose in lung cancer. Pathway analysis through MPP shows the alteration or disturbance in lactic acid, carbon dioxide and phosphoric acid involved in pyruvate metabolism and citric acid (TCA) cycle between controls and lung cancer. This is shown in Fig. S4 of supplementary material.

### Fatty acid triacylglycerol and ketone body metabolism

Alterations of several lipids metabolism are often observed in lung cancer samples, including over-expression of fatty acid synthase (FAS). Comparatively high levels of fatty acids, including palmitic acid, octadecanoic acid, stearic acid and cholesterol were found in the plasma samples of lung cancer as compared to controls. FAS serves to store the energy derived from carbohydrate metabolism. Fatty acids are esterified to phospholipids, such as phophatidylcholine^28^. They are activated to acyl-CoA in a 2-step reaction, forming diacylglycerides with glycerol 3-phosphate. These diacylglycerides then react with CDP choline to form phosphatidylcholine. Pathway analysis through MPP shows the alteration in phosphoric acid, palmitate, carbon dioxide, glycerol and archidonic acid involved in fatty acid triacylglycerol and ketone body metabolism between controls and lung cancer as shown in Fig. S5 of supplementary material. Over expression of FAS has been observed in many lung cancers studies^10^,^11^,^29^. Experimental studies have indicated that various oncogenic signaling pathways lead to increased FAS expression^30^,^31^. Recently SREBP (Sterol Regulatory Element-Binding Protein, a transcription factor and is a direct target of PI3K/Akt and MAPK pathways) that regulates the lipid synthesis and uptake through up-regulation of key enzymes of lipogenesis^32^,^33^. High content of glucose may be due to the high requirement of energy of lung cancer cells which results in carbohydrate metabolism and lipogenesis to provide the energy in the form of glucose.

### GPCR downstream signaling

In cancer cells (lung, gastric, colorectal, pancreatic and prostatic cancers) abnormal expression of GPCRs and/or their ligands has been observed^34^,^35^. Pathway analysis shows increase in phosphoric acid, glycerol and arachidonic acid levels in lung cancer, involved in GPCR downstream signaling pathway derived from endocannabinoids anandamide (AEA) and 2-arachidonoyl glycerol (2-AG). The resulting altered pattern of receptor expression is shown in Fig. S6 of supplementary material. This consequently leads to changes in fatty acid synthesis and glucose utilization^36^.

### ABC family protein mediated transport

ABC transporters are membrane proteins which generate energy from ATP hydrolysis to actively transport a variety of compounds across the membrane, including ions, sugars, amino acids, lipids, toxins and anticancer drugs. ABC transporters are involved in tumor resistance. ABCB1 or MDR1 P-glycoprotein are involved in lipid transport which is their main function^37^. Pathway analysis shows the alteration of phosphoric acid and cholesterol involved in ABC family protein mediated transport, as shown in Fig. S7 of the supplementary material.

### Bile acid and bile salt metabolism

Bile acids are steroidal amphipathic molecules, derived from the catabolism of cholesterol. The catabolism of cholesterol to bile acids is an important route for the elimination of cholesterol from the body, accounting for approximately 50% of cholesterol eliminated daily. Bile acids are involved in signal transduction pathways that regulate apoptosis^38^. Pathway analysis shows the alternation of phosphoric acid and cholesterol, involved in bile acid and bile salt metabolism, as shown in Fig. S8 of the supplementary material.

Up-regulation of acidic environment (decrease pH) in cancer cells is common due to production of lactic acid. Our experimental data shows high level of lactic acid, phosphoric acid and benzoic acid in lung cancer patients, as compared to controls. Acidic environment of cancer typically results in necrosis or apoptosis through p53 and caspase-3-dependent mechanisms^39^. Consequently, up-regulation of glycolysis requires resistance to apoptosis or up-regulation of membrane transporters to maintain pH. These changes may result in a malignant phenotype and facilitate local invasion and metastasis formation^39^.

### Concluding remarks

Our study has shown that GC-MS-based metabolite profiling of blood plasma using 2D-C18 fractionation approach followed by chemometric analyes is able to identify biomarker metabolites which can significantly differentiate lung cancer from three control groups (healthy non-smokers, smokers and COPD) with high sensitivity (96.2%) and specificity (92.05%). The two groups, i.e. lung cancer (LC) and COPD are much close to each other (dissimilarity level of only 0.206 by cluster analysis). Elevated levels of almost all the fatty acids, glucose and acids were found in lung cancer patients, in comparison to the controls. Generally, glycolysis increased in cancer but in this study high level of glucose was found in lung cancer samples as compare to controls. However, high level of glucose in lung cancer does not show the decrease in glycolysis as lactic acid is also up-regulated in lung cancer. From the pathway analysis, it was concluding that glycolysis results in the breakdown of glucose, but several reactions may be responsible for the increased levels of glucose in lung cancer like gluconeogenesis, carbohydrate metabolism and lipogenesis to provide the energy in the form of glucose. Up regulation of acidic environment (decrease pH) and alterations of several lipid metabolism favors the lung cancer growth. A promising finding is the newly built model based on thirty two significantly metabolites which accurately classifies lung cancer and controls on external validation. Unfortunately, only 37% of the metabolites were characterized and their pathways are correlated. Identification of unknown metabolites with high resolution can increase human metabolome and ultimately help in biomarker identification of lung cancer.

## Supplementary Material

Supplementary Information Supplementary Dataset 1

## Figures and Tables

**Table 1:** Experimental subject description - healthy non-smokers and smokers

Age range (years)	Number of Samples (codes)		
	Healthy males	Healthy females	Smokers
20–30	20 (HMPG1 1–20)	20 (HFPG1 1–20)	33 (SMPG1-1–33)
30–40	10 (HMPG2 1–10)	10 (HFPG2 1–10)	19 (SMPG2-1–19)
40–50	10 (HMPG3 1–10)	10 (HFPG3 1–10)	24 (SMPG3-1–24)
Above 50	10 (HMPG4 1–10)	10 (HFPG4 1–10)	24 (SMPG4-1–24)

**Table 2:** Experimental subject description- lung cancer patients

Type of cancer	Number of Samples (codes)
Squamous cell carcinoma	10 (SqLC1–SqLC11)
Adenocarcinoma	12 (AdLC1–AdLC12)
Small cell carcinoma	16 (SmLC1–SmLC13)
Non Small cell carcinoma (not categorized)	10 (NSCLC1–NSC LC8)
Not categorized	52 (LC1–LC50)

**Table 3:** List of metabolites (32 entities) that are distinguished between three controls, healthy non-smokers (NS), smokers (S), chronic obstructive pulmonary disease (COPD) and lung cancer (LC) at p < 0.001 and fold change >3 and CV < 25%

(a) Compounds or (b) Base peak (*m/z*)	R.T (mins)	p-value	Log FC (S VS NS)	Log FC (COPD VS NS)	Log FC (LC VS NS)	CV (NS)	CV (S)	CV (COPD)	CV (LC)
Lactic acid^a^	6.547	0.001	−0.75066	−0.75066	15.01397	6.059	0	0	0.940
Phosphoric acid^a^	9.298	4.46 × 10^−34^	−1.43 × 10^−06^	−2.15 × 10^−06^	11.38233	0	0	0	1.392
Benzoic acid^a^	8.925	0.001	−0.38841	−0.38841	14.88933	7.348	0	0	1.040
Naphthalene^a^	14.913	4.98 × 10^−23^	−7.88417	−7.61842	5.263484	1.524	0	8.659	1.042
d-Glucose^a^	17.000	0.001	−0.80834	−0.80834	16.77236	6.226	0	0	0.914
Altrose^a^	17.180	0.001	−0.77384	−0.77384	15.65283	5.159	0	0	0.988
Palmitic acid^a^	18.082	0.001	−0.86307	−0.56074	18.47669	5.338	0	8.660	0.881
Octadecanoicacid^a^	19.248	0.001	24.80116	22.96099	13.00723	0	0.423	0.598	1.504
Stearic acid^a^	19.876	0.001	−0.8934	−0.61384	18.4489	5.203	0	8.659	0.878
1-Propene^a^	22.708	5.55 × 10^−21^	−4.91746	−16.7018	−2.27579	0.706	1.739	0	0.960
Cholesterol^a^	27.099	0	−0.80642	−0.2636	17.42233	5.173	0	6.446	1.119
79.0^b^	6.466	7.83 × 10^−16^	−0.6038909	0.6598923	−17.717346	1.053	1.286	1.190	0
221.0^b^	6.627	1.03 × 10^−28^	−0.36994314	−0.36994243	10.512385	7.348	0	0	1.135
138.0^b^	7.211	6.1 × 10^−25^	0.31311202	0.79443276	10.497155	0	8.123	6.579	1.464
192.0 b	9.245	0.001	−1.5639739	−1.0004983	18.191345	3.979	0	706.215	0.659
57.0^b^	9.430	6.32 × 10^−16^	−0.49987864	−2.316762	7.907245	2.990	3.839	0	1.723
179.0^b^	10.948	1.72 × 10^−33^	−0.7623167	−0.76231694	10.744249	5.363	0	0	1.216
77.0^b^	15.036	2.93 × 10^−21^	13.092134	0.574327	9.222518	0	1.111	7.658	1.769
77.0^b^	15.138	6.60 × 10^−39^	−1.306344	15.365888	−1.3063436	4.165	0	0.838	0
312.0^b^	15.956	0.001	−0.42208862	−0.13931417	19.562046	7.348	0	8.659	0.899
129^b^	20.794	0.001	−3.3007922	−3.3007927	15.475743	2.493	0	0	0.619
91.0^b^	21.747	8.32 × 10^−43^	−8.707808	−11.9619255	11.135013	1.164	2.823	0	0.527
91.0^b^	21.799	0.001	21.44686	24.984108	1.0096989	0	0.471	0.444	5.049
61.0^b^	23.255	8.98 × 10^−36^	13.969654	0.55730176	0	0	0.975	6.101	0
104.0^b^	23.364	2.28 × 10^−35^	−2.6220374	−2.6220374	10.5647	2.660	0	0	0.898
91.0^b^	23.396	0.001	−6.757048	−6.7570477	15.541145	1.693	0	0	0.529
91.0^b^	23.452	0.001	20.630585	19.240364	9.54 × 10^−07^	0	0.531	0.653	0
91.0^b^	23.560	0.001	−4.3524594	−4.352461	16.166733	2.181	0	0	0.554
179.0^b^	24.409	0.001	−0.7441251	−0.74412465	13.964065	5.201	0	0	0.842
179.0^b^	25.822	0.001	−1.5109181	−1.5109181	16.547422	3.942	0	0	0.805
91.0^b^	26.856	0.001	−1.19 × 10^−06^	−1.67 × 10^−06^	15.379847	0	0	0	0.891

^a^Identified metabolites.

^b^Unidentified metabolites.

## References

[b1] Ganti A. K. & Mulshine J. L. Lung Cancer Screening. The oncologist 11, 481–487, 10.1634/theoncologist.11-5-481 (2006). 16720848

[b2] Wardwell N. R. & Massion P. P. Novel strategies for the early detection and prevention of lung cancer. Seminars in oncology 32, 259–268 (2005). 15988680 10.1053/j.seminoncol.2005.02.009

[b3] Ferlay J. S. I. *et al.* GLOBOCAN 2012 v1.0, Cancer Incidence and Mortality Worldwide: IARC CancerBase No. 11 [Internet]. Lyon, France: International Agency for Research onCancer (2013). Available at: http://globocan.iarc.fr/Pages/fact_sheets_population.aspx. (Accessed:12 June 2014).

[b4] Hassanein M. *et al.* The state of molecular biomarkers for the early detection of lung cancer. Cancer prevention research (Philadelphia, Pa.) 5, 992–1006, 10.1158/1940-6207.capr-11-0441 (2012). 22689914 PMC3723112

[b5] Ramirez J. L. *et al.* Methylation patterns and K-ras mutations in tumor and paired serum of resected non-small-cell lung cancer patients. Cancer letters 193, 207–216 (2003). 12706879 10.1016/s0304-3835(02)00740-1

[b6] Musharraf S. G. *et al.* Comparison of plasma from healthy nonsmokers, smokers, and lung cancer patients: pattern-based differentiation profiling of low molecular weight proteins and peptides by magnetic bead technology with MALDI-TOF MS. Biomarkers: biochemical indicators of exposure, response, and susceptibility to chemicals 17, 223–230, 10.3109/1354750x.2012.657245 (2012). 22356277

[b7] Hori S. *et al.* A metabolomic approach to lung cancer. Lung Cancer 74, 284–292 (2011). 21411176 10.1016/j.lungcan.2011.02.008

[b8] Yang J. *et al.* High Performance Liquid Chromatography−Mass Spectrometry for Metabonomics: Potential Biomarkers for Acute Deterioration of Liver Function in Chronic Hepatitis B. Journal of Proteome Research 5, 554–561, 10.1021/pr050364w (2006). 16512670

[b9] Kami K. *et al.* Metabolomic profiling of lung and prostate tumor tissues by capillary electrophoresis time-of-flight mass spectrometry. Metabolomics 9, 444–453, 10.1007/s11306-012-0452-2 (2013). 23543897 PMC3608864

[b10] Rocha C. M. *et al.* Metabolic signatures of lung cancer in biofluids: NMR-based metabonomics of blood plasma. J Proteome Res 10, 4314–4324, 10.1021/pr200550p (2011). 21744875

[b11] Carrola J. *et al.* Metabolic signatures of lung cancer in biofluids: NMR-based metabonomics of urine. J Proteome Res 10, 221–230, 10.1021/pr100899x (2011). 21058631

[b12] Jordan K. W. *et al.* Comparison of squamous cell carcinoma and adenocarcinoma of the lung by metabolomic analysis of tissue-serum pairs. Lung Cancer 68, 44–50, 10.1016/j.lungcan.2009.05.012 (2010). 19559498 PMC2834857

[b13] Baeten K., Adriaensens P. & Stinissen P. inventors. Metabolic markers for diagnosing of cancer patent. World Intellectual Property Organization patent WO 2011128256 A1. Oct 20 2011.

[b14] Maeda J. *et al.* Possibility of multivariate function composed of plasma amino acid profiles as a novel screening index for non-small cell lung cancer: a case control study. BMC Cancer 10, 690 (2010). 21176209 10.1186/1471-2407-10-690 PMC3014908

[b15] Dong J. *et al.* Lysophosphatidylcholine profiling of plasma: discrimination of isomers and discovery of lung cancer biomarkers. Metabolomics 6, 478–488, 10.1007/s11306-010-0215-x (2010).

[b16] Guo Y. *et al.* Probing gender-specific lipid metabolites and diagnostic biomarkers for lung cancer using Fourier transform ion cyclotron resonance mass spectrometry. Clinica chimica acta; international journal of clinical chemistry 414, 135–141, 10.1016/j.cca.2012.08.010 (2012). 22906735

[b17] Fan T. *et al.* Altered regulation of metabolic pathways in human lung cancer discerned by (13) C stable isotope-resolved metabolomics (SIRM). Mol. Cancer 8, 41–59 (2009). 19558692 10.1186/1476-4598-8-41 PMC2717907

[b18] Xue R. *et al.* A serum metabolomic investigation on hepatocellular carcinoma patients by chemical derivatization followed by gas chromatography/mass spectrometry. Rapid Communications in Mass Spectrometry 22, 3061–3068 (2008). 18767022 10.1002/rcm.3708

[b19] Elizabeth J., Nordström A., Morita H. & Siuzdak G. From exogenous to endogenous: the inevitable imprint of mass spectrometry in metabolomics. J. Proteome Res. 6, 459–468 (2007). 17269703 10.1021/pr060505+

[b20] Niu Y. *et al.* Preliminary results of metabolite in serum and urine of lung cancer patients detected by metabolomics. Zhongguo fei ai za zhi = Chinese journal of lung cancer 15, 195–201, 10.3779/j.issn.1009-3419.2012.04.01 (2012). 22510503 PMC5999985

[b21] Phillips M. *et al.* Detection of lung cancer using weighted digital analysis of breath biomarkers. Clinica Chimica Acta 393, 76–84 (2008). 10.1016/j.cca.2008.02.021 PMC2497457 18420034

[b22] Poli D. *et al.* Determination of aldehydes in exhaled breath of patients with lung cancer by means of on-fiber-derivatisation SPME-GC/MS. Journal of chromatography. B, Analytical technologies in the biomedical and life sciences 878, 2643–2651, 10.1016/j.jchromb.2010.01.022 (2010). 20149763

[b23] Song G. *et al.* Quantitative breath analysis of volatile organic compounds of lung cancer patients. Lung Cancer 67, 227–231 (2010). 19409642 10.1016/j.lungcan.2009.03.029

[b24] Kischkel S. *et al.* Breath biomarkers for lung cancer detection and assessment of smoking related effects—confounding variables, influence of normalization and statistical algorithms. Clinica Chimica Acta 411, 1637–1644 (2010). 10.1016/j.cca.2010.06.005 20542019

[b25] O'Neill H. J., Gordon S. M., O'Neill M. H., Gibbons R. D. & Szidon J. P. A computerized classification technique for screening for the presence of breath biomarkers in lung cancer. Clinical chemistry 34, 1613–1618 (1988). 3042190

[b26] Musharraf S. G., Mazhar S., Siddiqui A. J., Choudhary M. I. & Atta ur R. Metabolite profiling of human plasma by different extraction methods through gas chromatography-mass spectrometry-an objective comparison. Anal Chim Acta 804, 180–189, 10.1016/j.aca.2013.10.025 (2013). 24267080

[b27] Catovsky D. *et al.* A classification of acute leukaemia for the 1990s. Annals of hematology 62, 16–21 (1991). 2031964 10.1007/BF01714978

[b28] Kuhajda F. P. Fatty acid synthase and cancer: new application of an old pathway. Cancer Res 66, 5977–5980, 10.1158/0008-5472.can-05-4673 (2006). 16778164

[b29] Menendez J. A. & Lupu R. Fatty acid synthase and the lipogenic phenotype in cancer pathogenesis. Nature reviews Cancer 7, 763–777, 10.1038/nrc2222 (2007). 17882277

[b30] Menendez J. A. *et al.* Inhibition of fatty acid synthase (FAS) suppresses HER2/neu (erbB-2) oncogene overexpression in cancer cells. Proceedings of the National Academy of Sciences of the United States of America 101, 10715–10720, 10.1073/pnas.0403390101 (2004). 15235125 PMC490000

[b31] Zhou W. *et al.* Fatty acid synthase inhibition activates AMP-activated protein kinase in SKOV3 human ovarian cancer cells. Cancer Res 67, 2964–2971, 10.1158/0008-5472.can-06-3439 (2007). 17409402

[b32] Krycer J. R., Sharpe L. J., Luu W. & Brown A. J. The Akt-SREBP nexus: cell signaling meets lipid metabolism. Trends in endocrinology and metabolism: TEM 21, 268–276, 10.1016/j.tem.2010.01.001 (2010). 20117946

[b33] Yang Y. A., Han W. F., Morin P. J., Chrest F. J. & Pizer E. S. Activation of fatty acid synthesis during neoplastic transformation: role of mitogen-activated protein kinase and phosphatidylinositol 3-kinase. Experimental cell research 279, 80–90 (2002). 12213216 10.1006/excr.2002.5600

[b34] Heasley L. E. Autocrine and paracrine signaling through neuropeptide receptors in human cancer. Oncogene 20, 1563–1569, 10.1038/sj.onc.1204183 (2001). 11313903

[b35] Rozengurt E. Neuropeptides as growth factors for normal and cancerous cells. Trends in endocrinology and metabolism: TEM 13, 128–134 (2002). 11893527 10.1016/s1043-2760(01)00544-6

[b36] Naughton S. S., Mathai M. L., Hryciw D. H. & McAinch A. J. Fatty Acid modulation of the endocannabinoid system and the effect on food intake and metabolism. International journal of endocrinology 2013, 361895, 10.1155/2013/361895 (2013). 23762050 PMC3677644

[b37] Wu C. P., Calcagno A. M. & Ambudkar S. V. Reversal of ABC drug transporter-mediated multidrug resistance in cancer cells: evaluation of current strategies. Current molecular pharmacology 1, 93–105 (2008). 19079736 10.2174/1874467210801020093 PMC2600768

[b38] St-Pierre M. V., Kullak-Ublick G. A., Hagenbuch B. & Meier P. J. Transport of bile acids in hepatic and non-hepatic tissues. The Journal of experimental biology 204, 1673–1686 (2001). 11316487 10.1242/jeb.204.10.1673

[b39] Gatenby R. A. & Gillies R. J. A microenvironmental model of carcinogenesis. Nature reviews Cancer 8, 56–61, 10.1038/nrc2255 (2008). 18059462

