# Supplementary material for: Plasma Metabolite Profiling and Chemometric Analyses of Lung Cancer along with Three Controls through Gas Chromatography-Mass Spectrometry
Source: Sci Rep. 2015 Feb 25;5:8607. doi: 10.1038/srep08607 (PMC4339805; doi:10.1038/srep08607)
Supplement: Supplementary Information — Supplementary Dataset 1 [file srep08607-s1.docx]

Supplementry Information

**Plasma Metabolite Profiling and Chemometric Analyses of Lung Cancer along with Three Controls through Gas Chromatography-Mass Spectrometry**

**Syed Ghulam Musharraf ^1,2^ Shumaila Mazhar^2^ Muhammad Iqbal Choudhary^1,2,3^ Nadeem Rizi^4^ and Atta-ur-Rahman^1,2^*

^1^Dr. Panjwani Center for Molecular Medicine and Drug Research, International Center for Chemical and Biological Sciences, University of Karachi, Karachi-75270, Pakistan.

^2^H.E.J. Research Institute of Chemistry, International Center for Chemical and Biological Sciences, University of Karachi, Karachi-75270, Pakistan.

^3^Department of Chemistry, College of Science, King Saud University, Riyadh-1145,

Saudi Arabia

^4^Jinnah Postgraduate Medical Center, Karachi, Pakistan

*Corresponding author. Tel.: +92 021-34824924; 4819010; fax: +92 021-34819018-9.

E-mail address: [musharraf1977@yahoo.com](mailto:musharraf1977@yahoo.com)

Supplementry Information


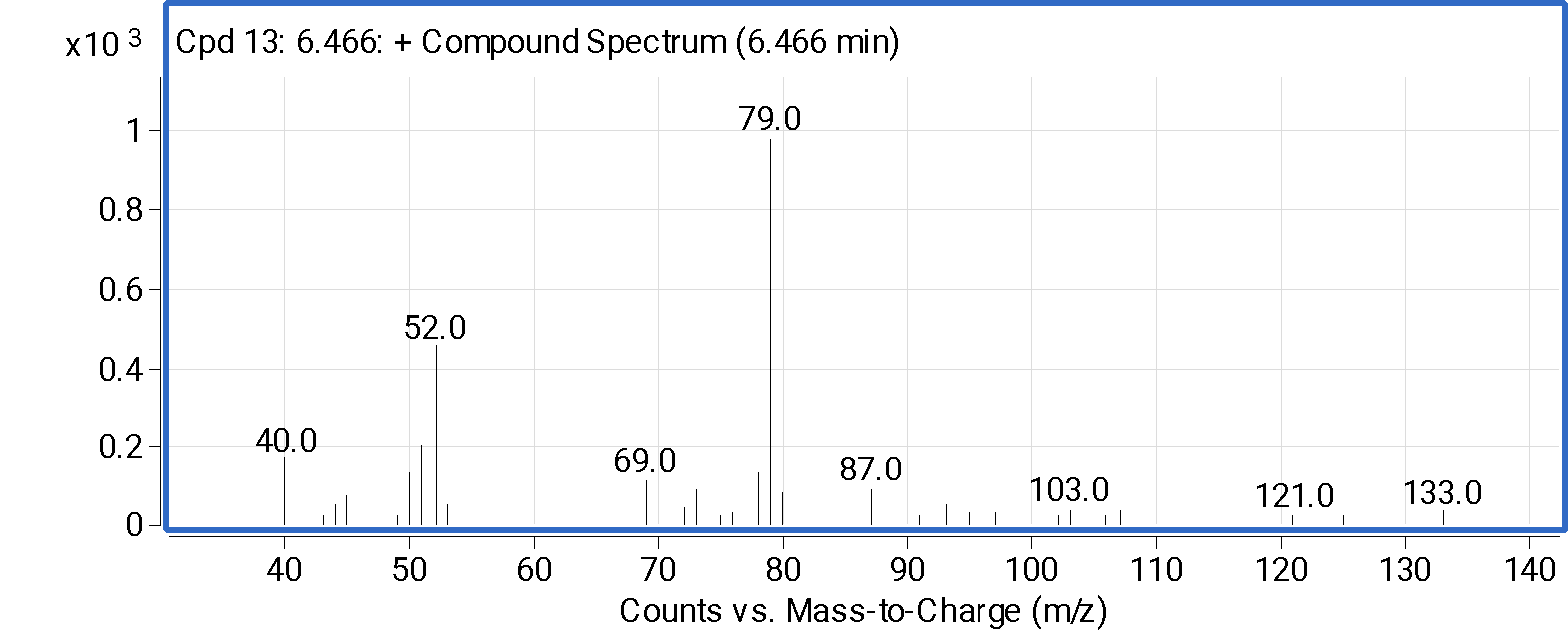


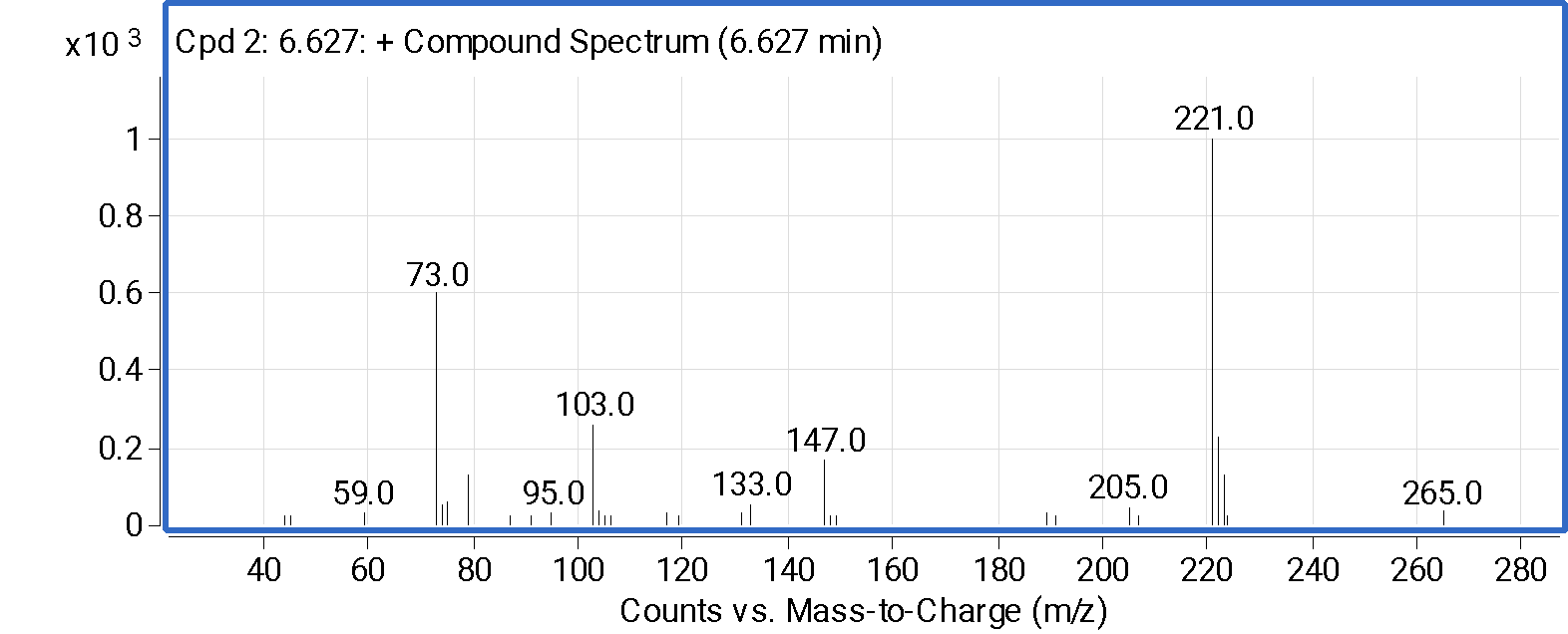


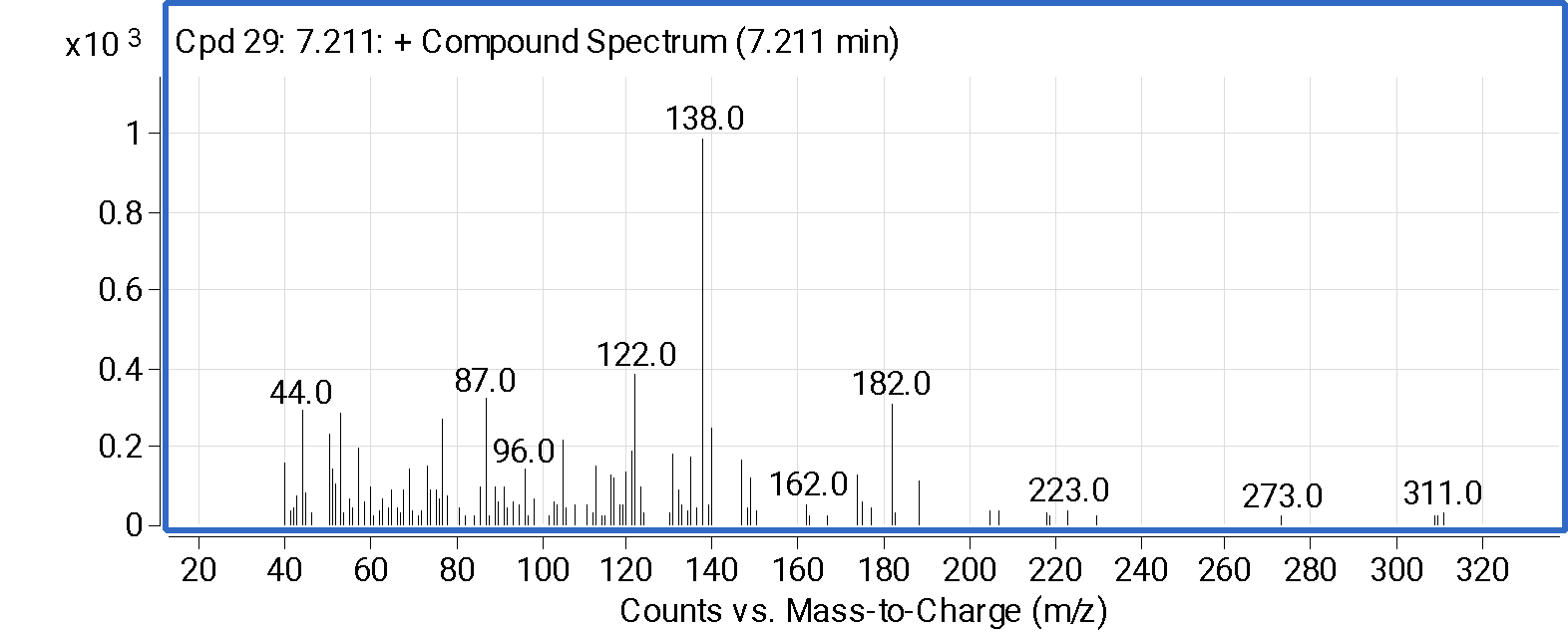


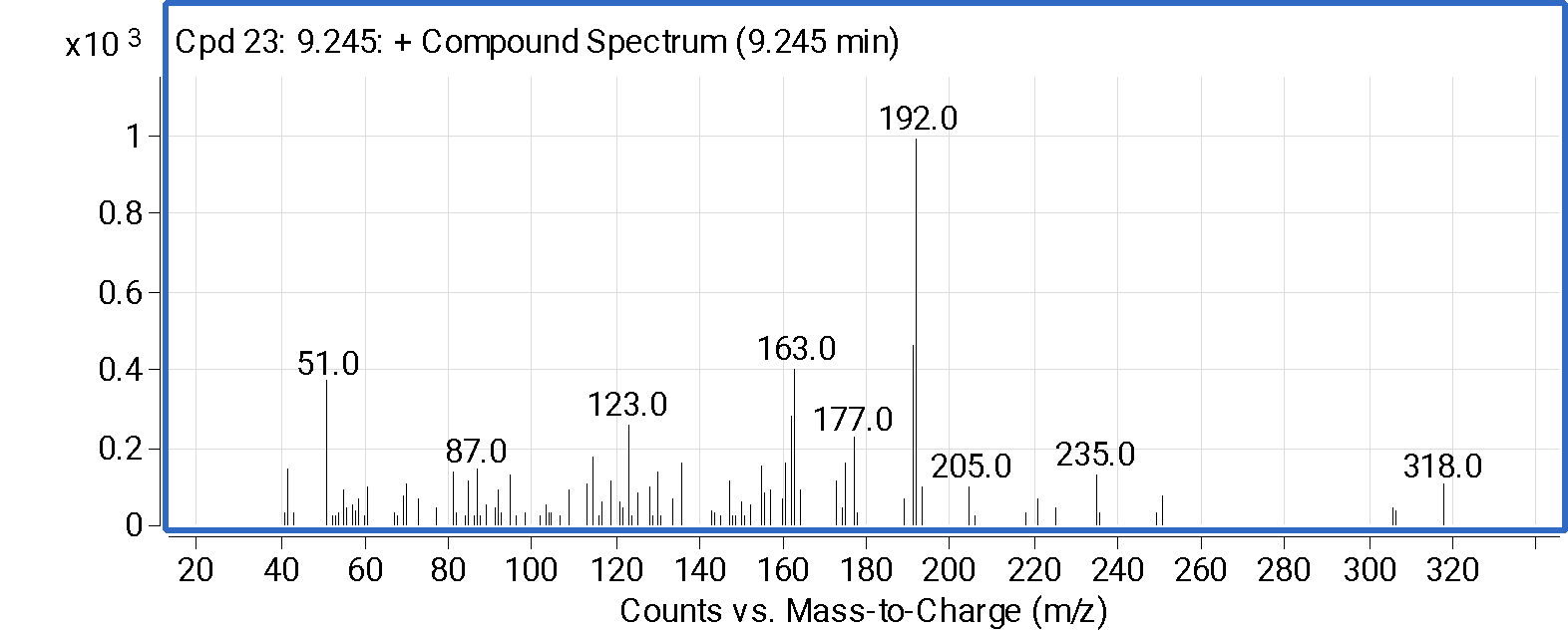


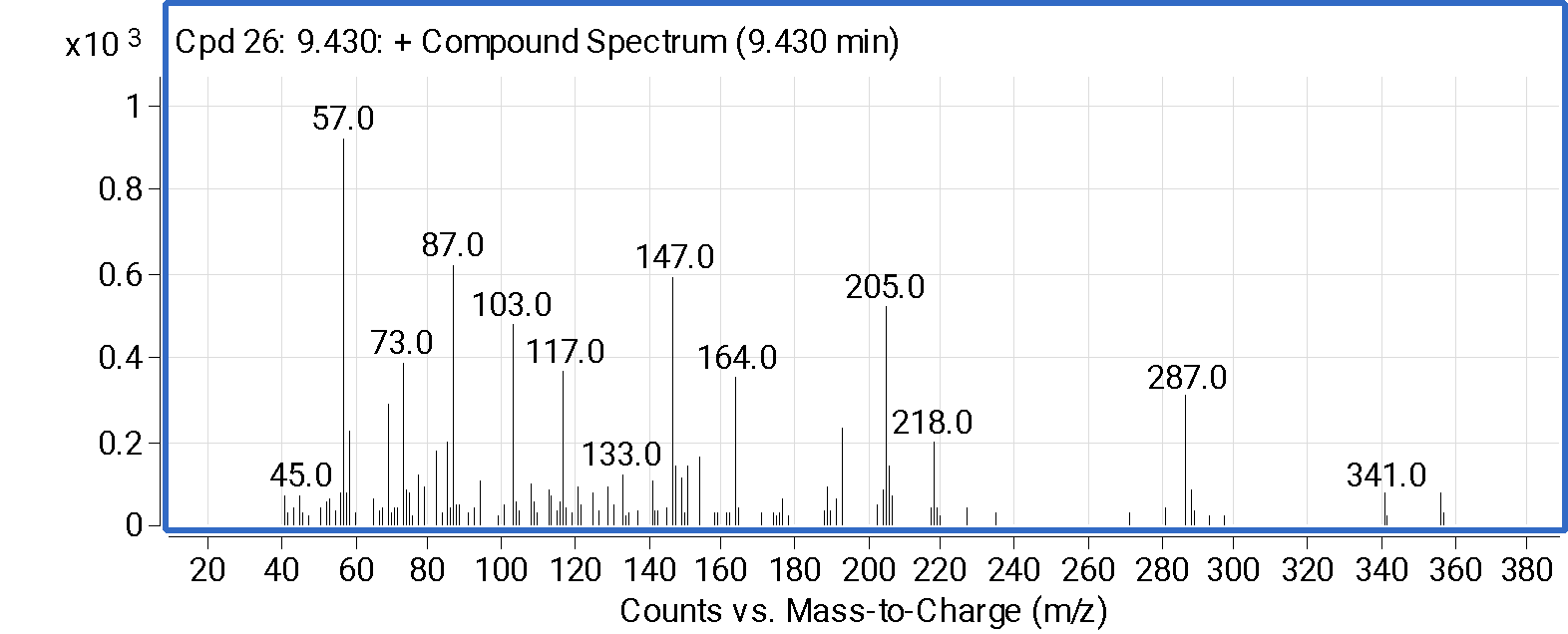


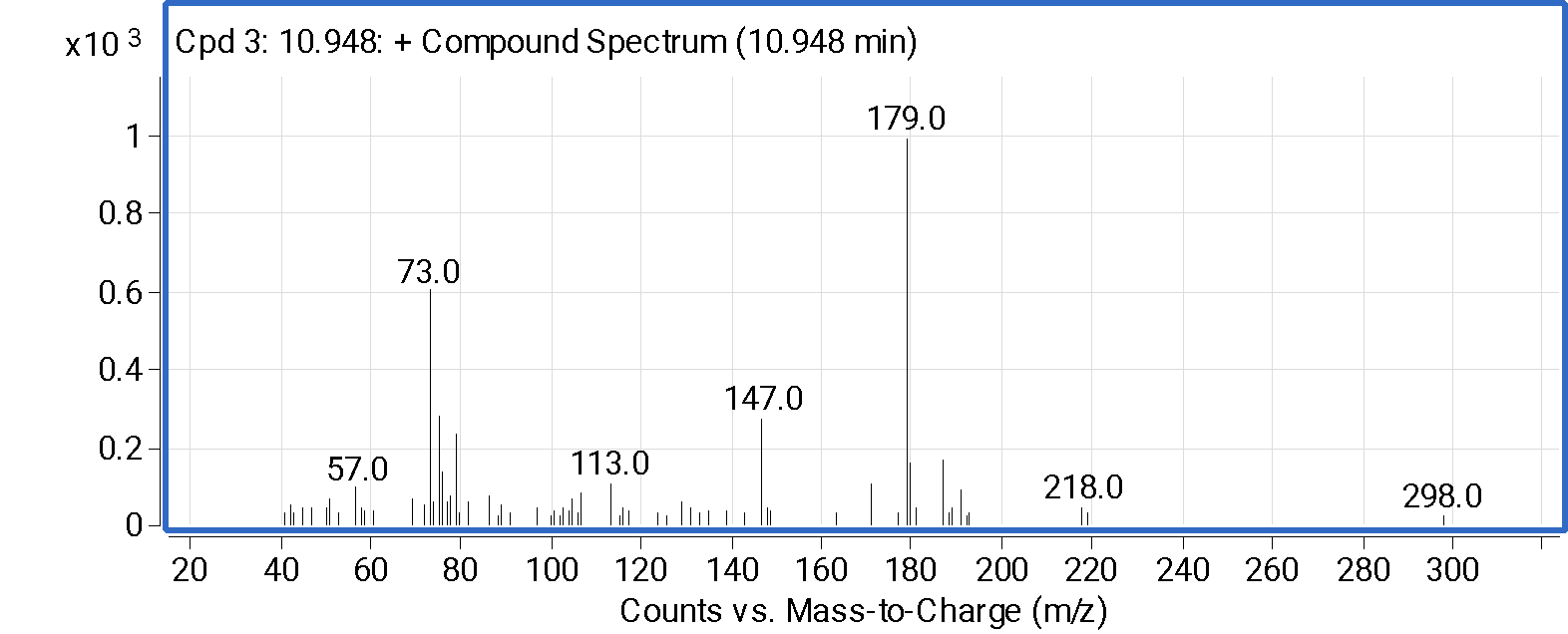


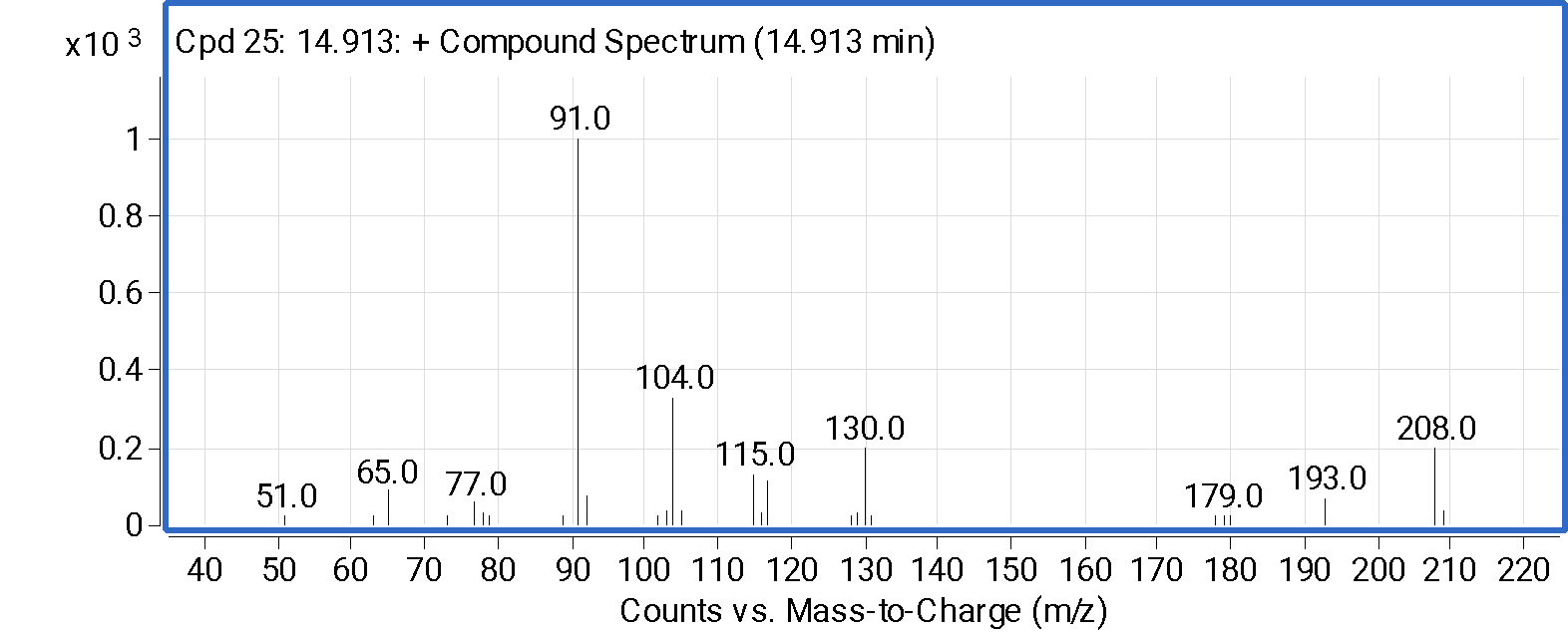


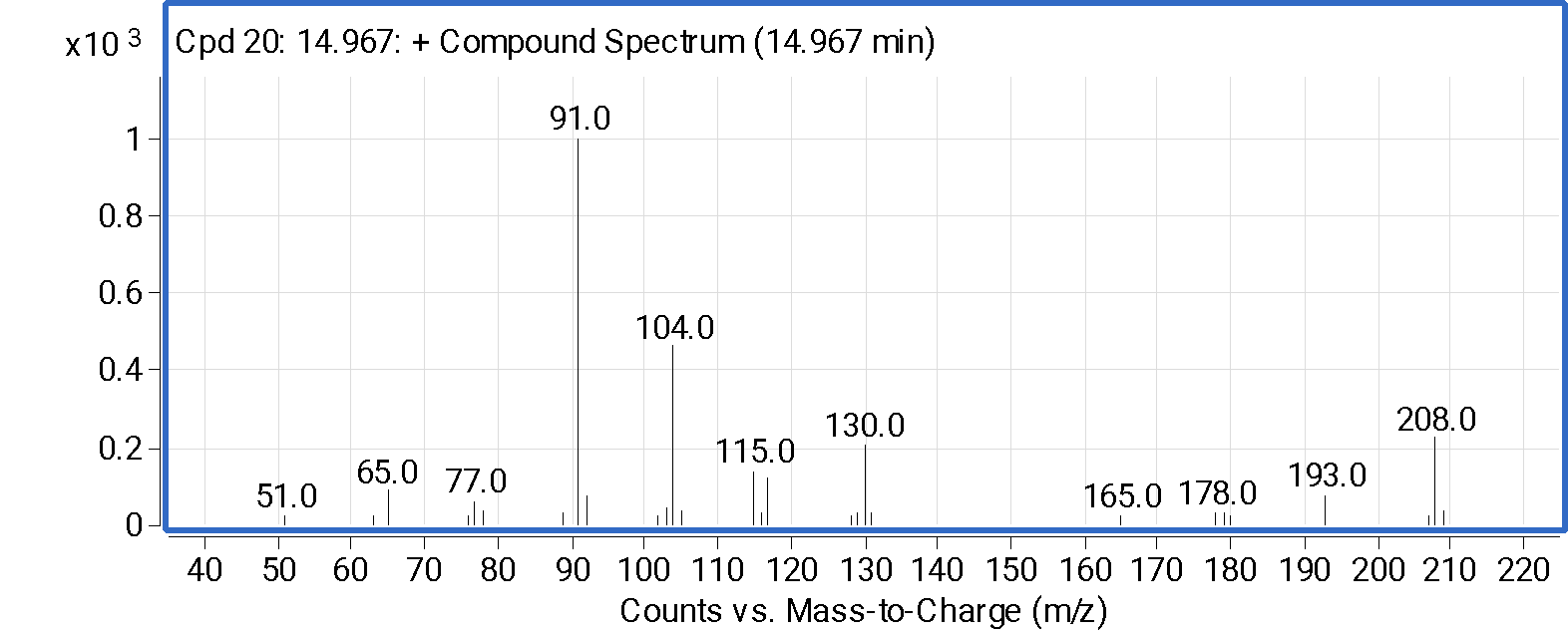


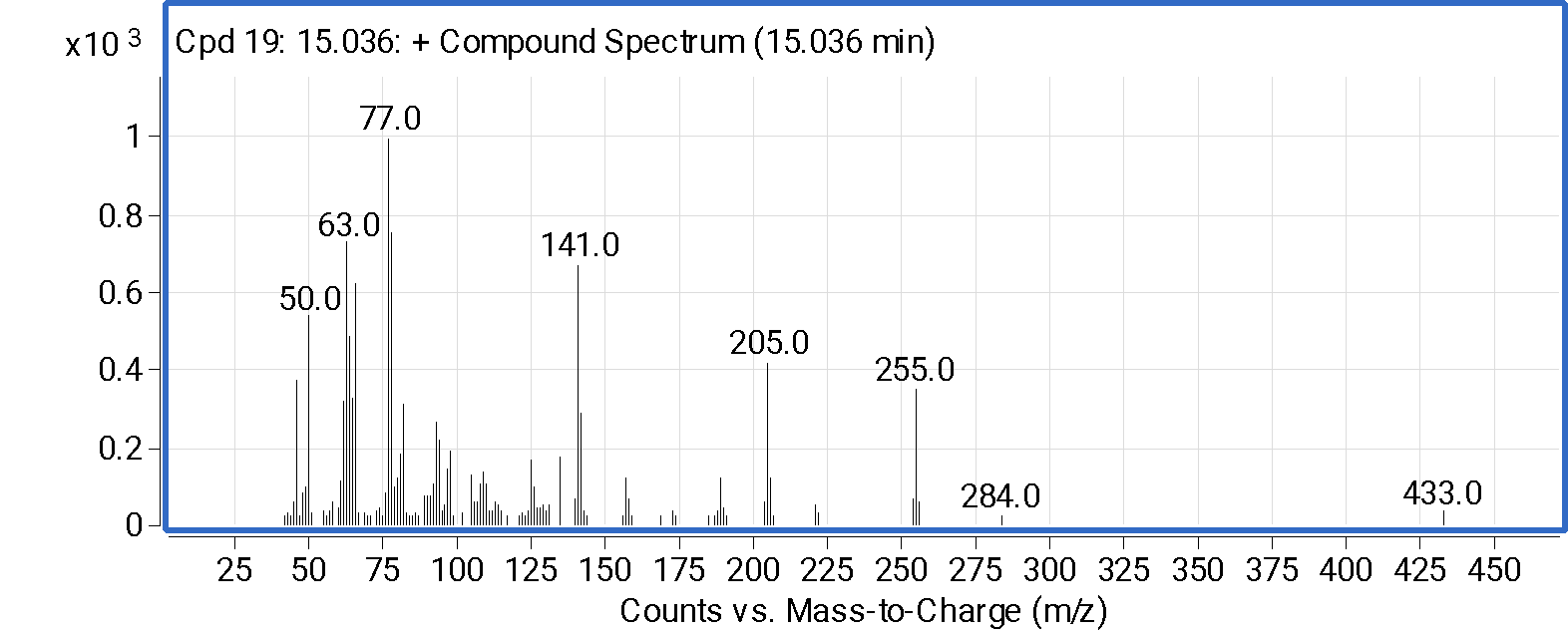


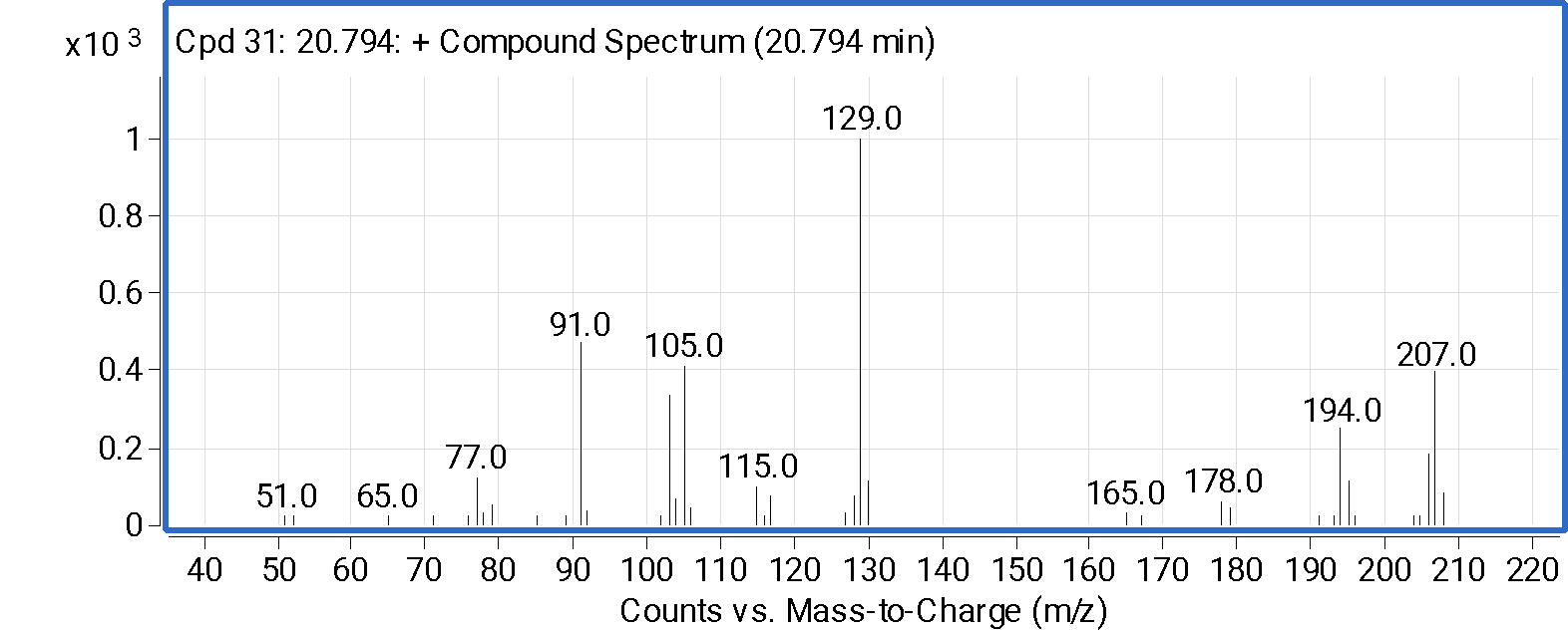


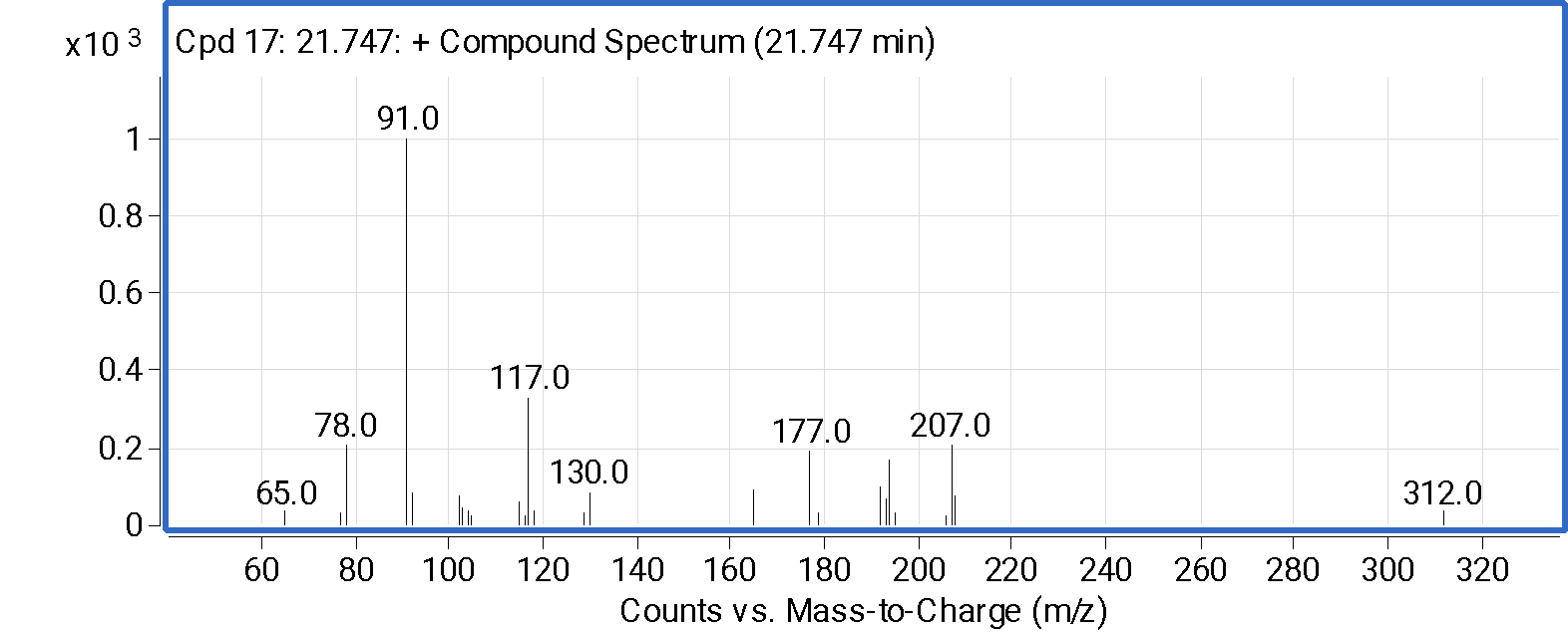


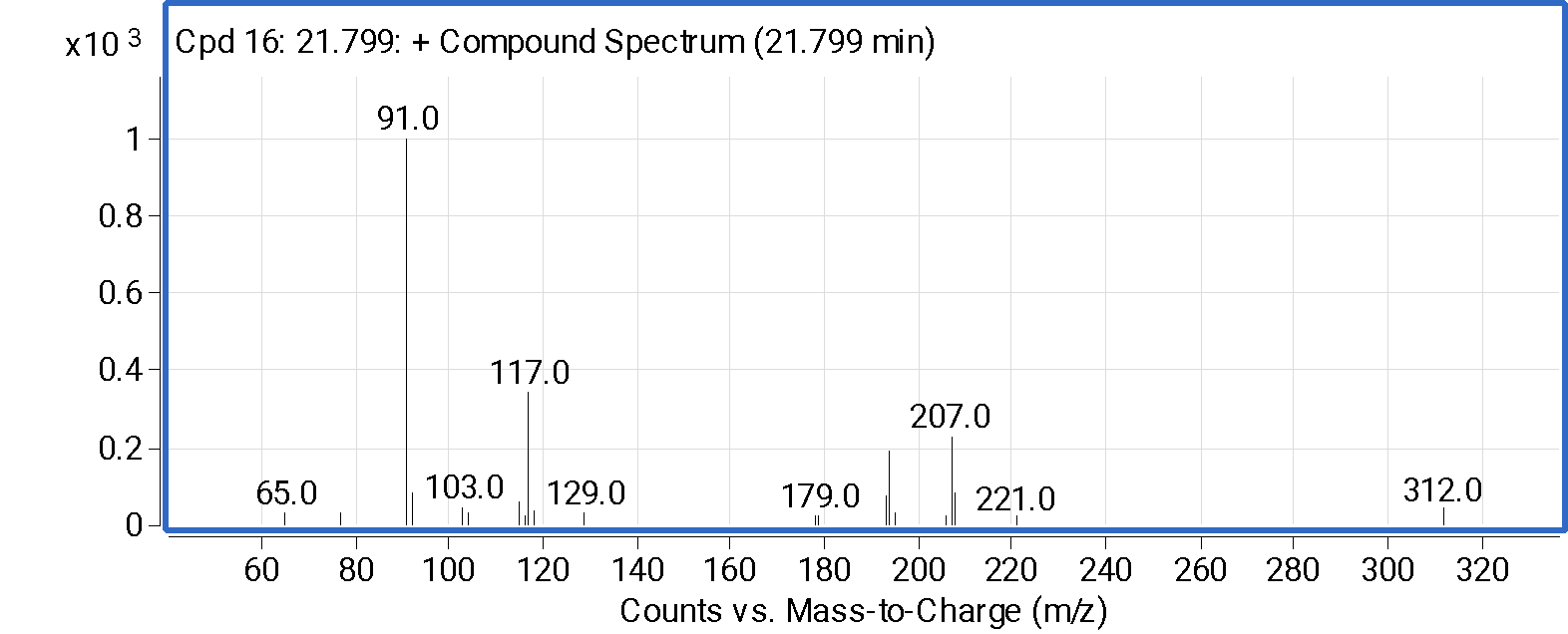


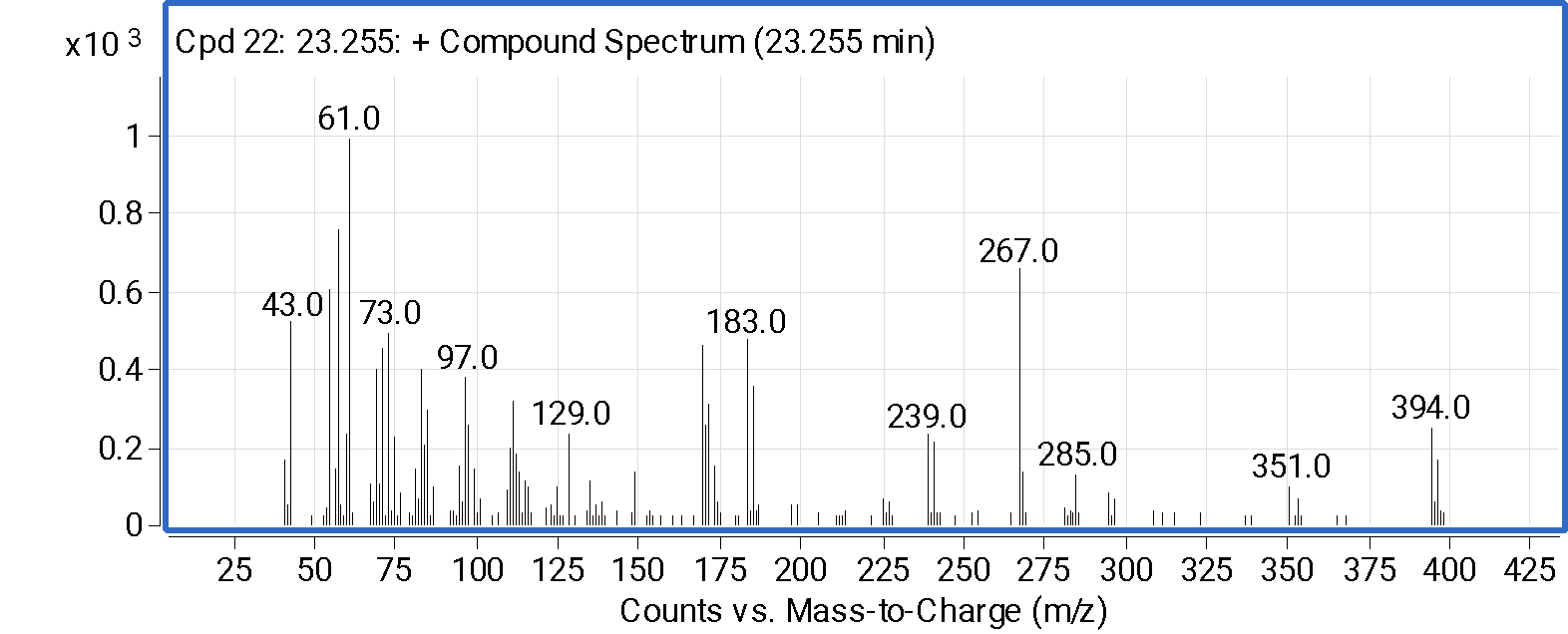


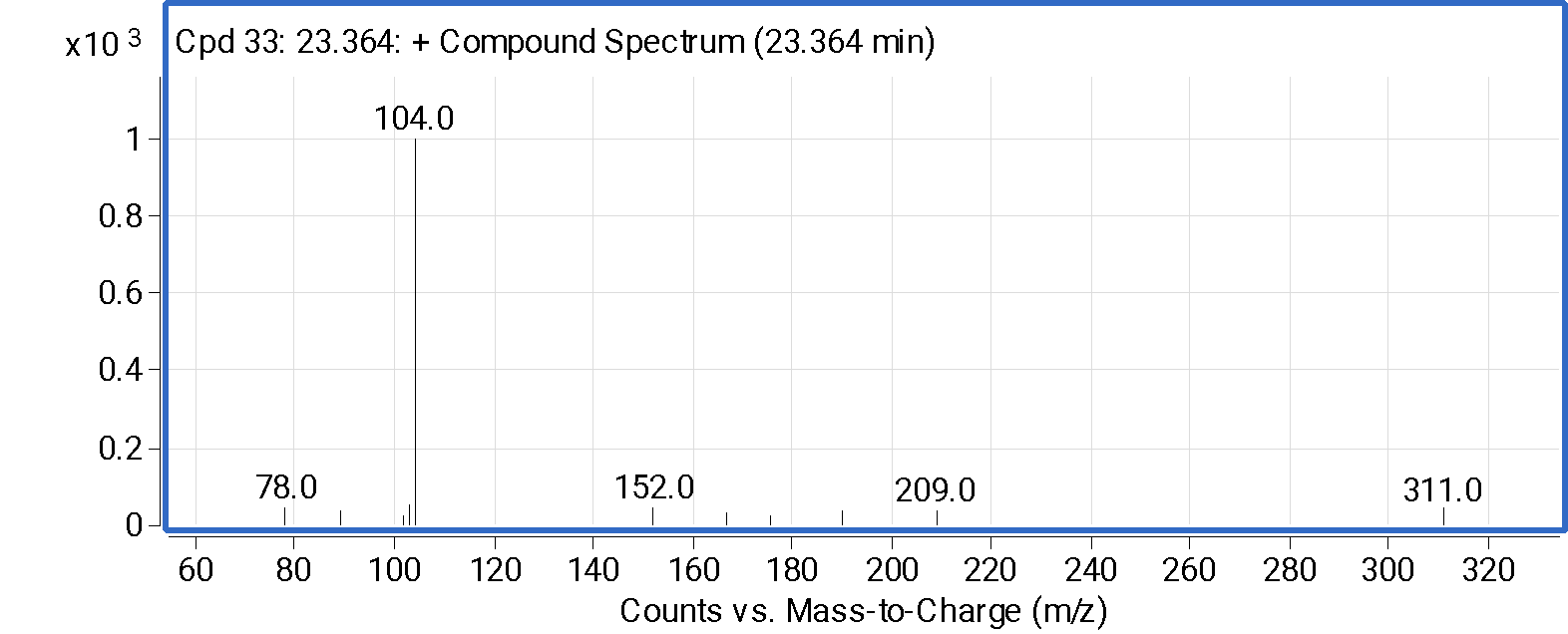


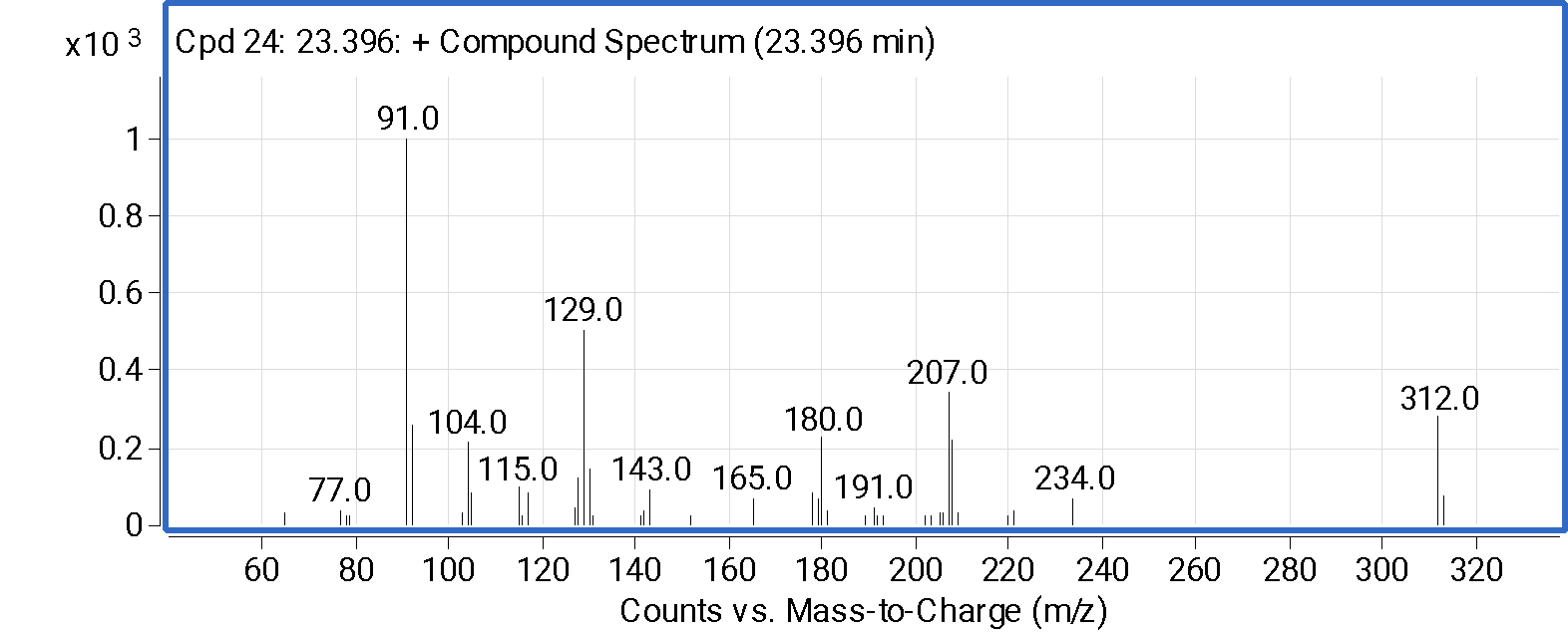


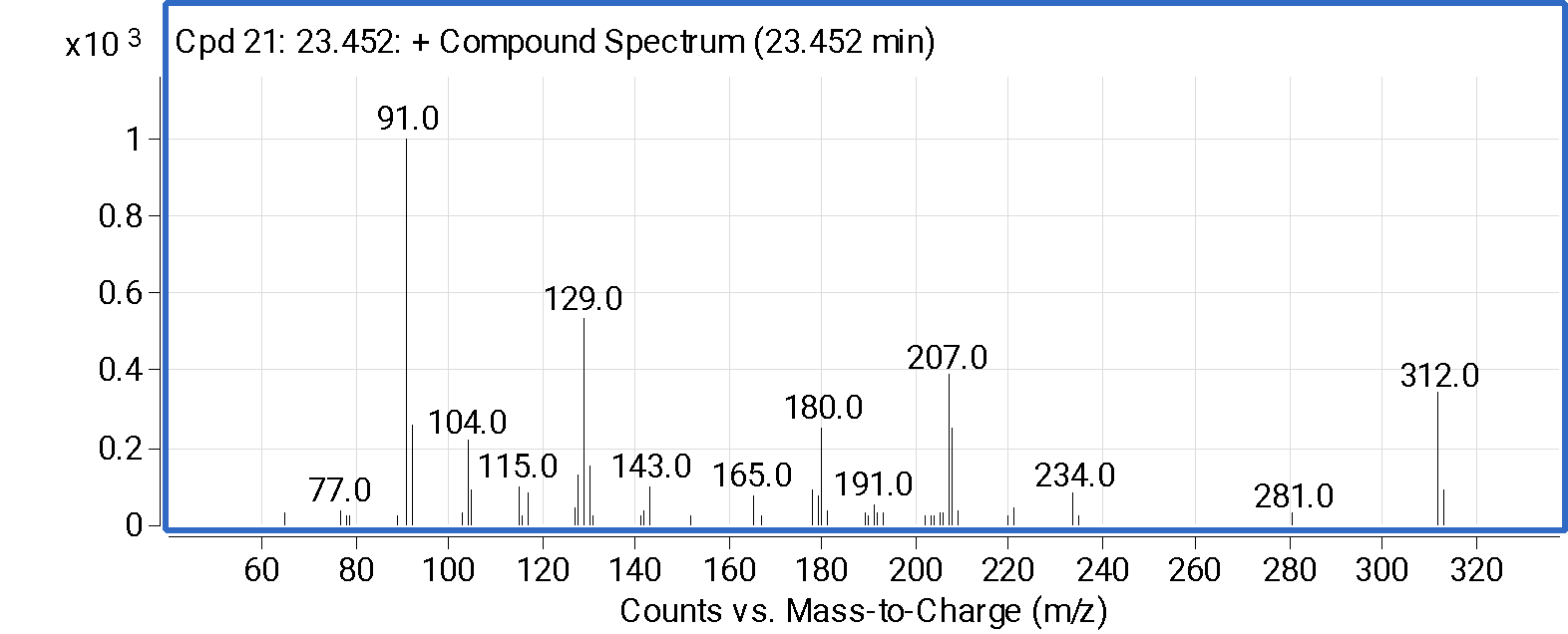


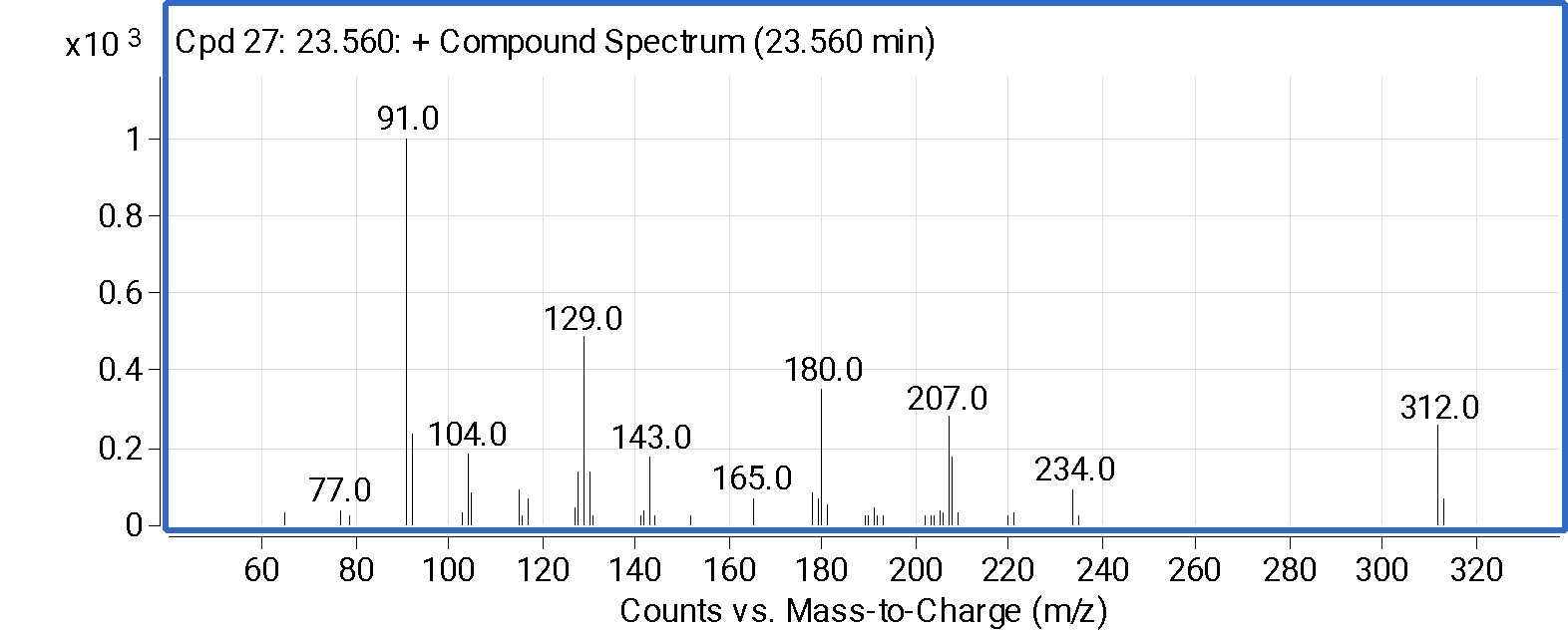


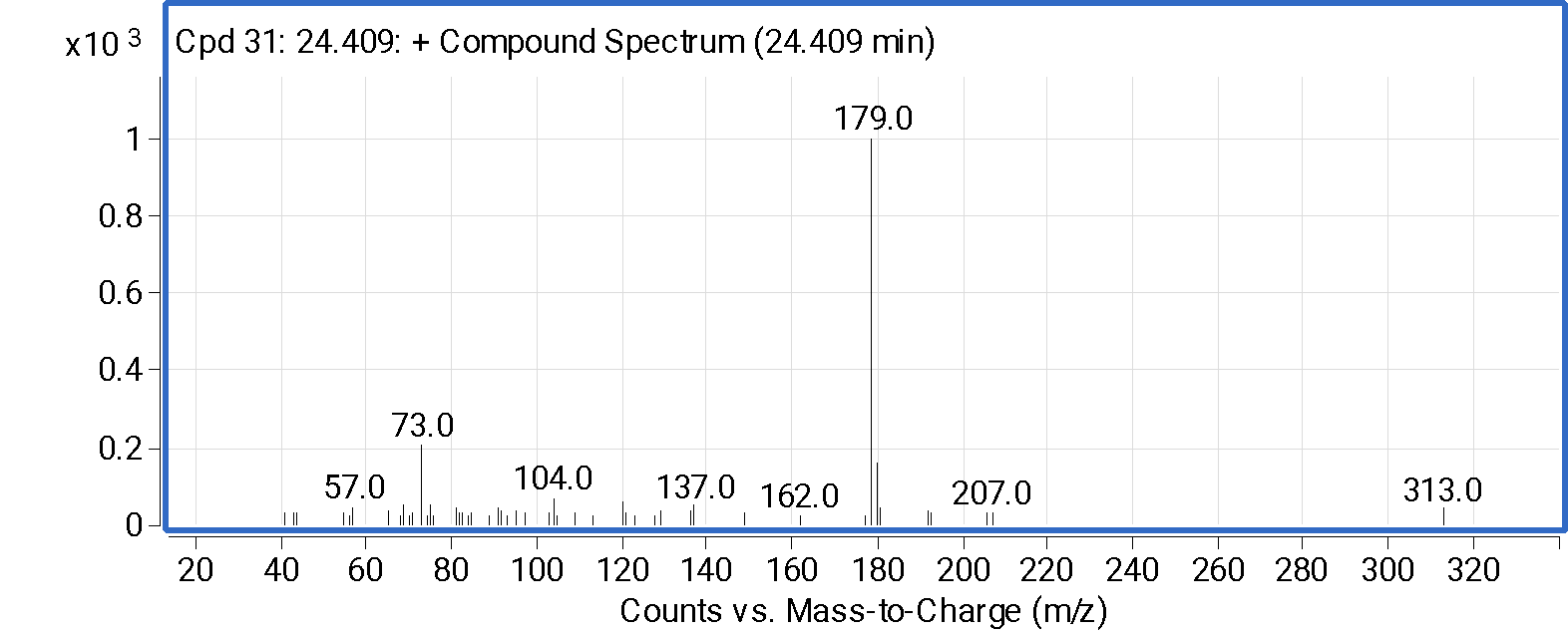


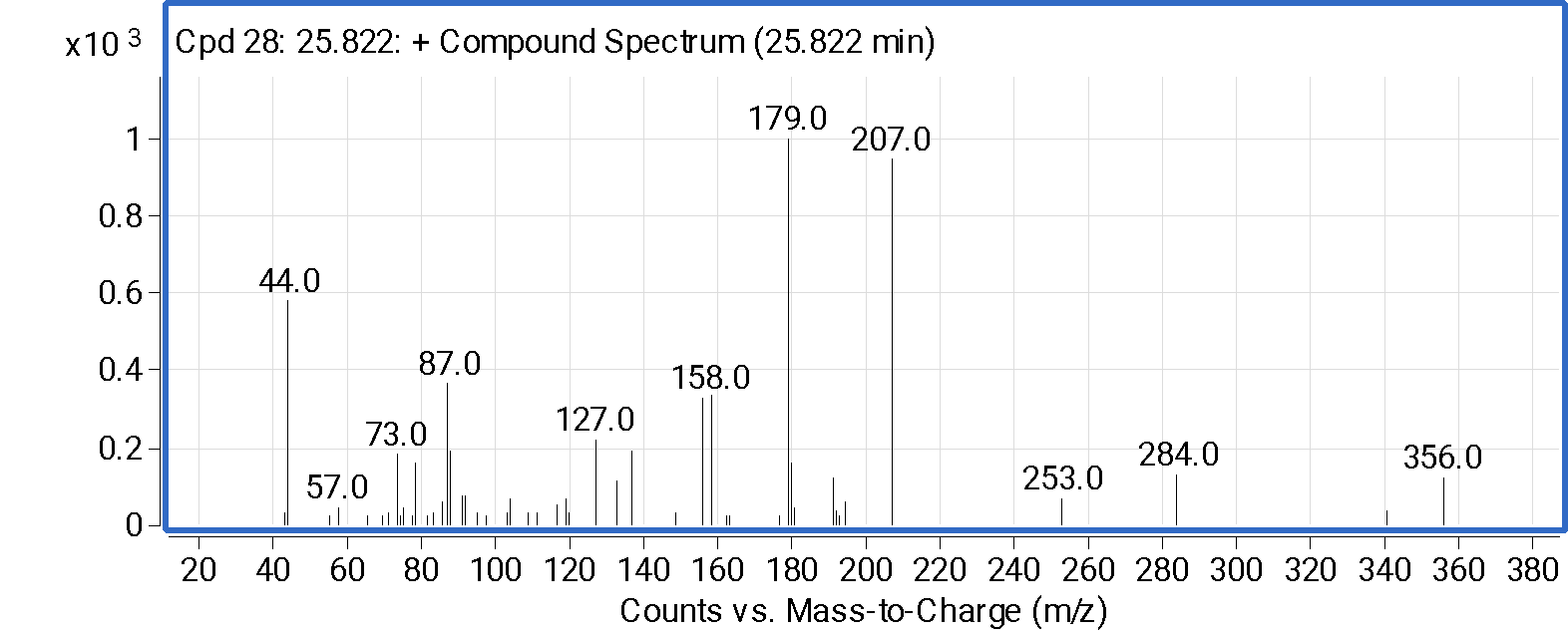


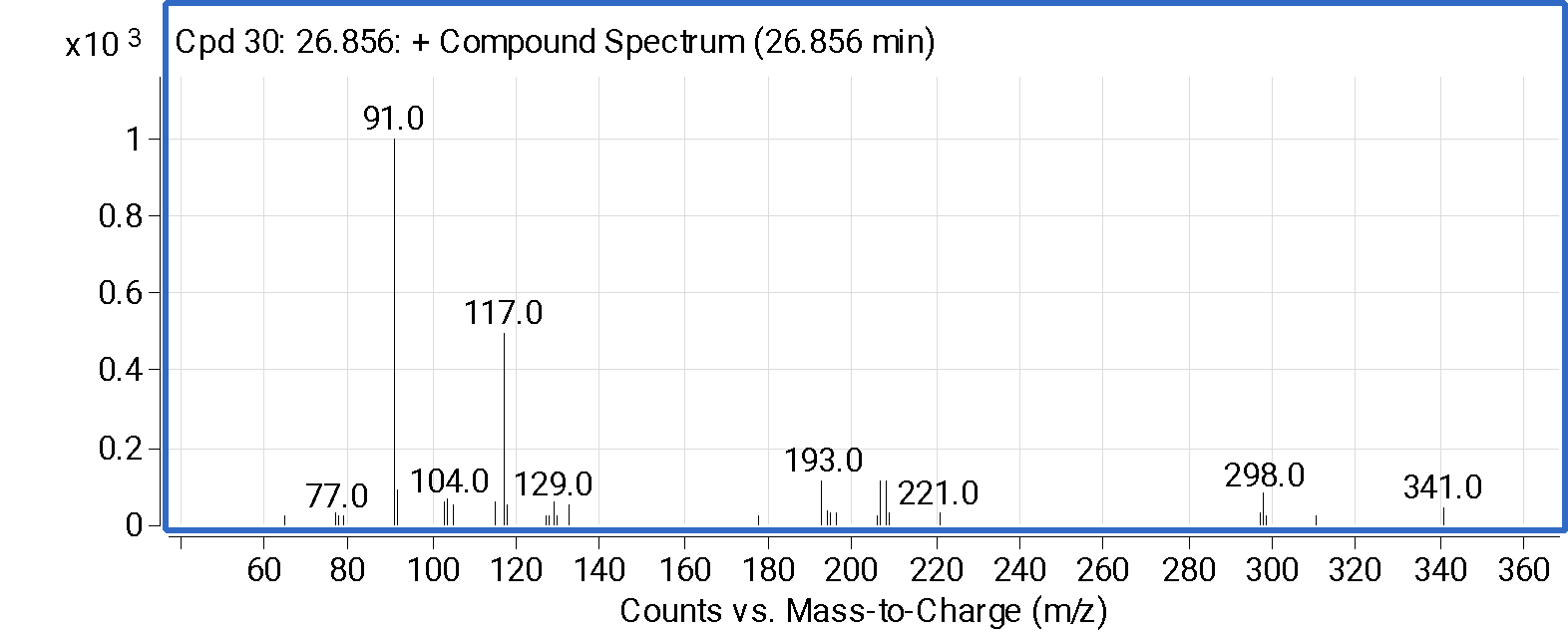


**Fig. S1.** The EI/MS spectra of unidentified compounds that are statistically differentially expressed between three controls, healthy non-smokers (NS), smokers (S), chronic obstructive pulmonary disease (COPD) and lung cancer (LC)


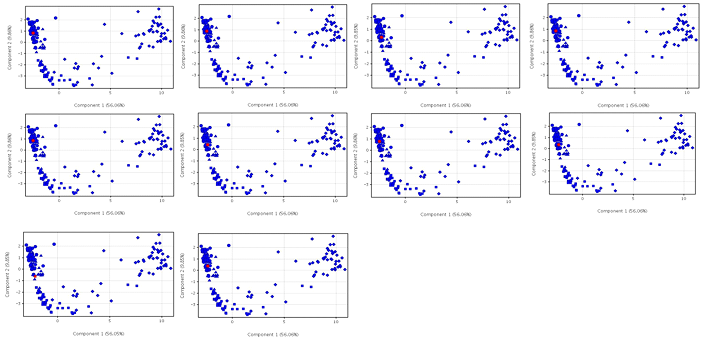


Sample **1-10** of COPD


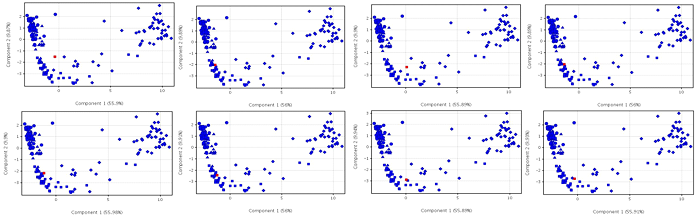


Sample 1-**8** of Healthy non-smoker


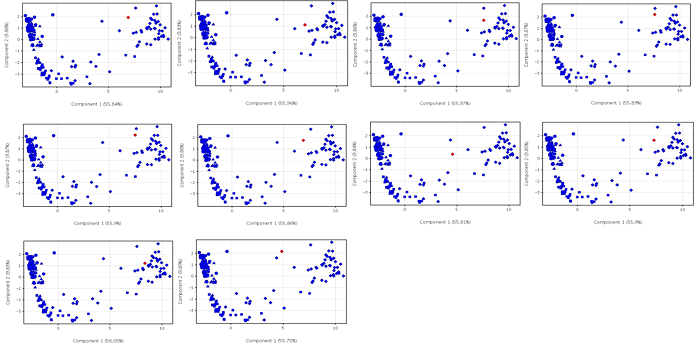


Sample **1-10** of Lung cancer


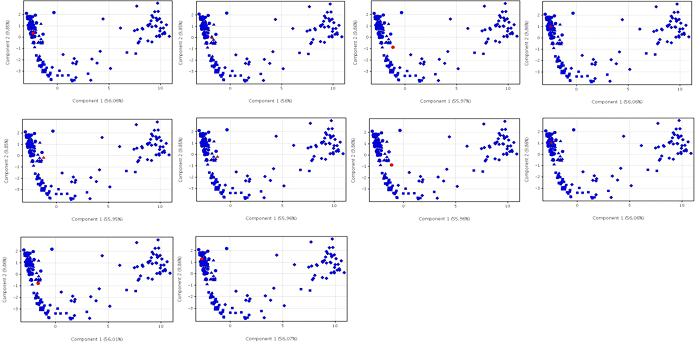


Sample 1-**10** of Smoker


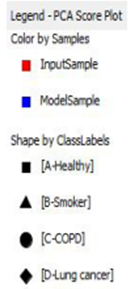


**Fig. S2.** Model generated PCA scores scatter plots discriminating among three controls and lung cancers based on the thirty two significance metabolites data and classify the 38 input samples.


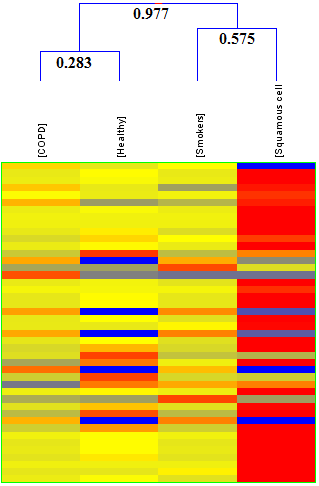

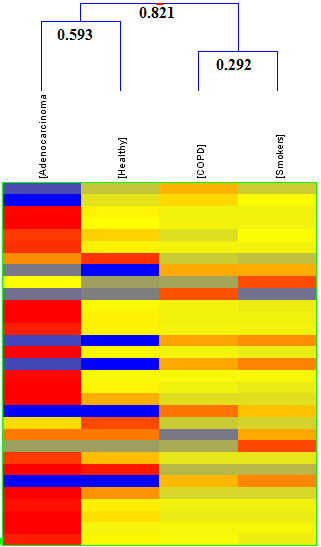


1. **(C)**
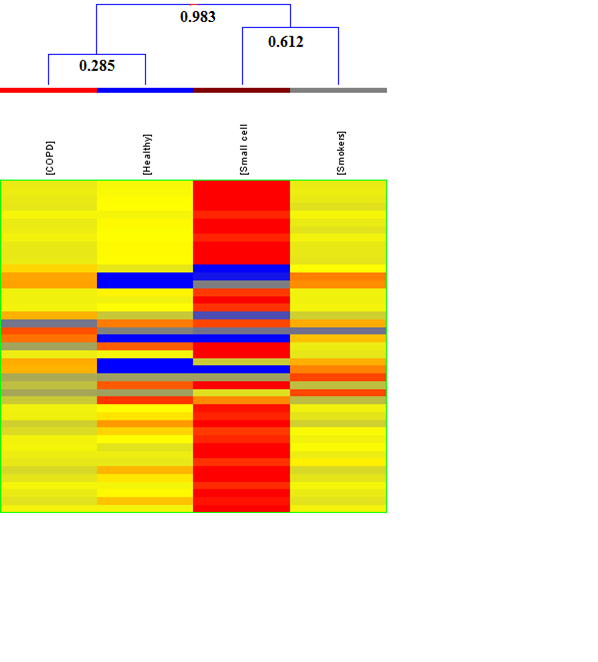

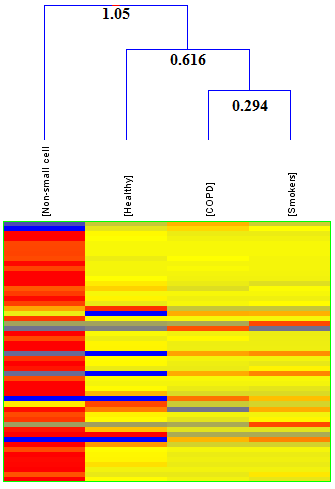

2. **(D)**

**Fig.S3.** Comparison of histological subgroup of lung cancer with control groups of samples i.e. (A) healthy non-smokers (NS), smokers (S), Chronic Obstructive Pulmonary Disease (COPD) and Squamous cell Lung Cancer (SqLC) (B) healthy non-smokers (NS), smokers (S), Chronic Obstructive Pulmonary Disease (COPD) and Small cell Lung Cancer (SmLC) (C) healthy non-smokers (NS), smokers (S), Chronic Obstructive Pulmonary Disease (COPD) and Adenocarcinoma Lung Cancer (AdLC) (D) healthy non-smokers (NS), smokers (S), Chronic Obstructive Pulmonary Disease (COPD) and Non-small Cell Lung Cancer (NSCLC) patients using normalized intensities of thirty two significance metabolites. The dendrogram was produced by applying a hierarchical clustering algorithm (Pearson’s uncentered-absolute distance metric, Complete Linkage).


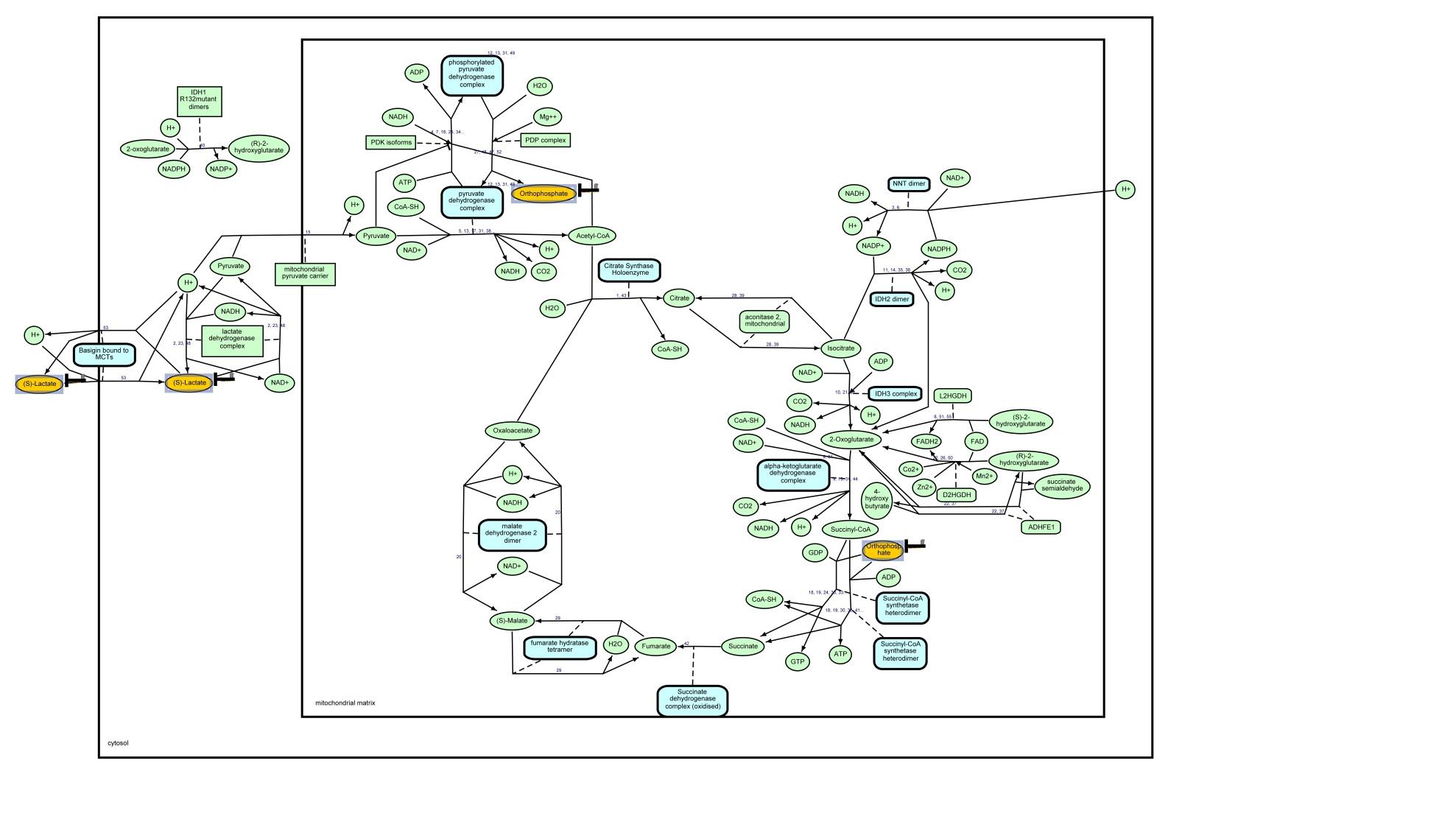


**Fig. S4**. Pyruvate metabolism and citric acid (TCA) cycle, alter metabolites are shown with yellow highlighted color between controls and lung cancer using 32 statistically differentiae metabolites.


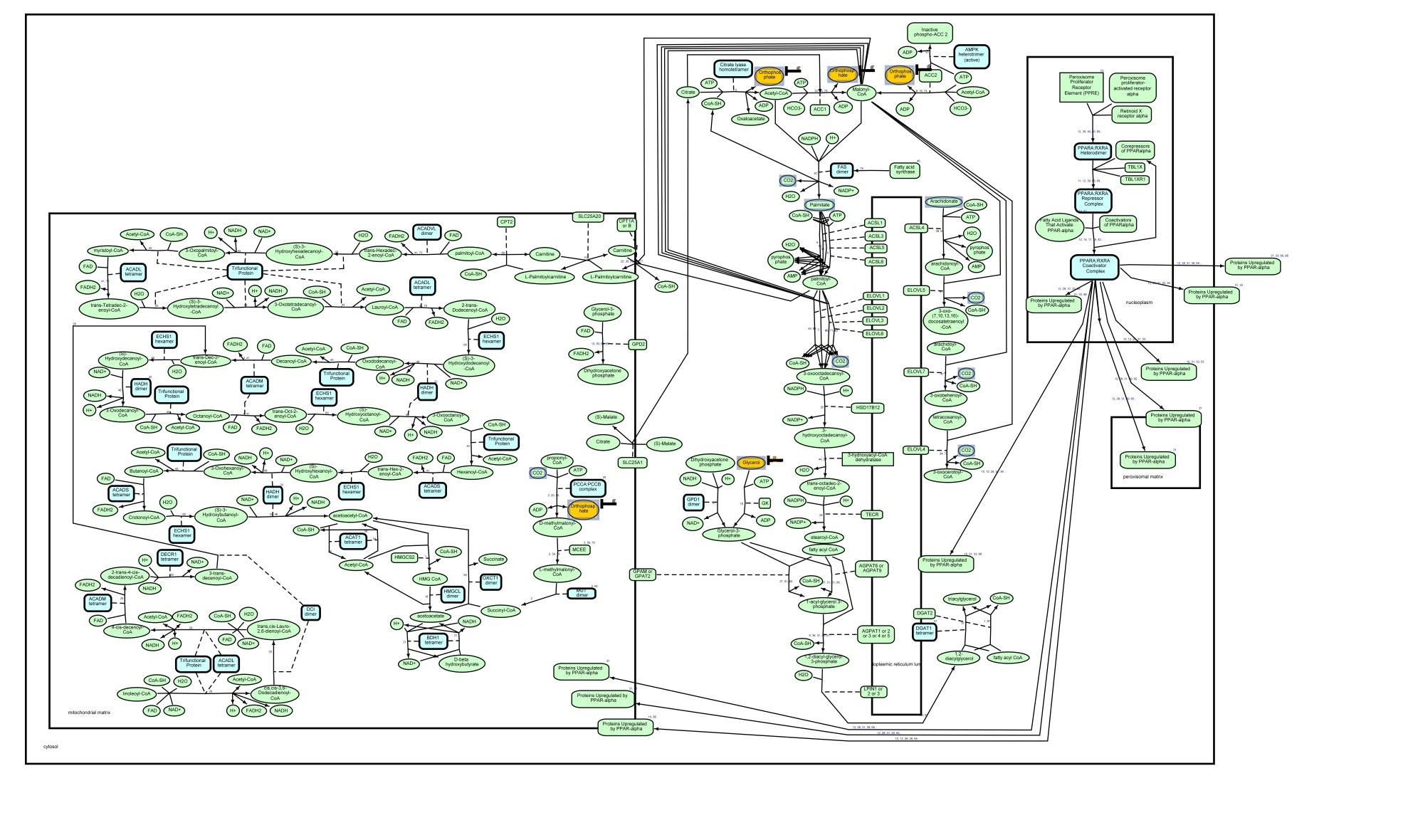


**Fig. S5**. Fatty acid triacylglycerol and ketone body metabolism, alter metabolites are shown with yellow highlighted color between controls and lung cancer using 32 statistically differentiae metabolites.


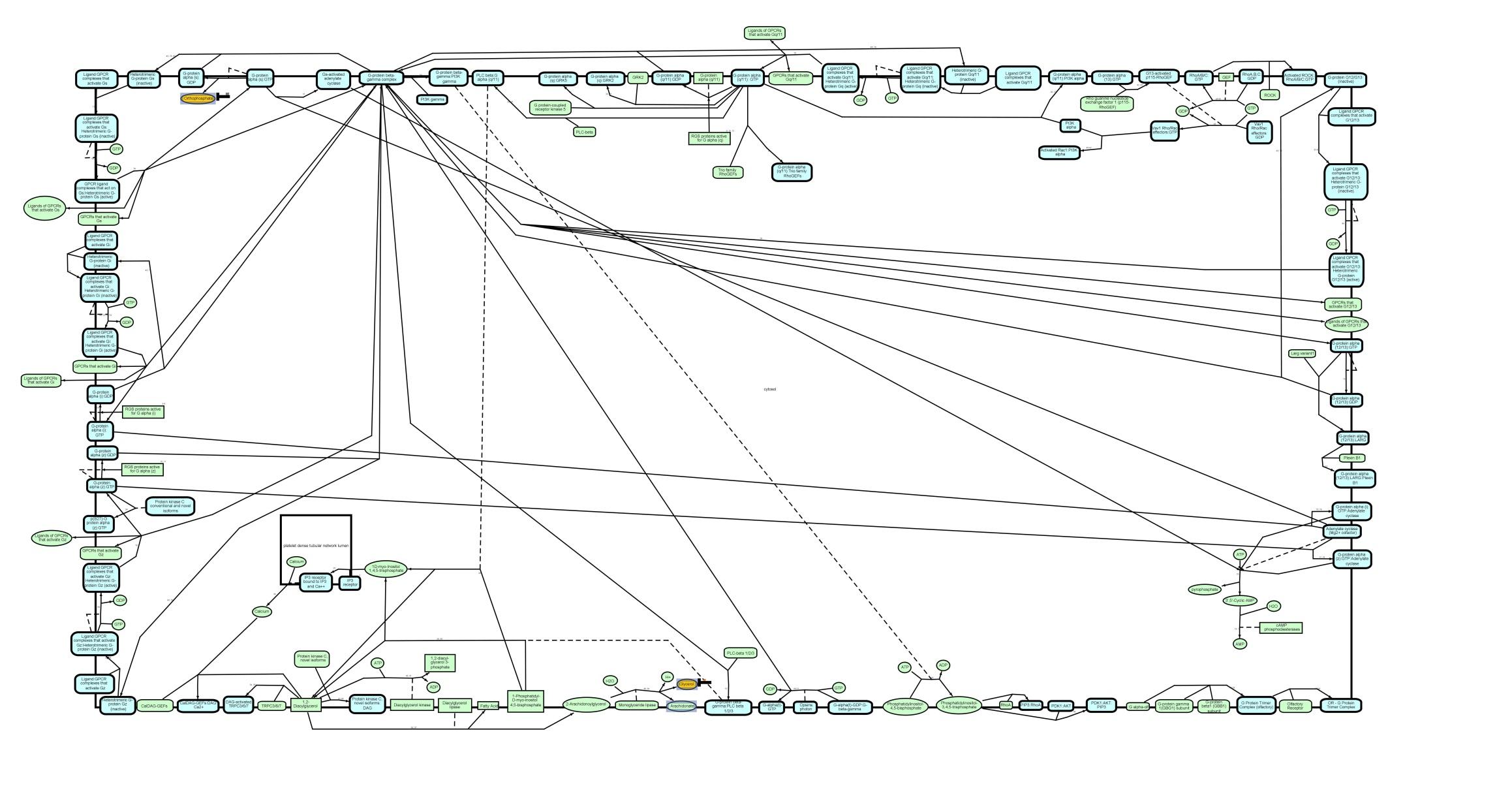
**Fig. S6**. GPCR downstream signaling, alter metabolites are shown with yellow highlighted color between controls and lung cancer using 32 statistically differentiae metabolites.


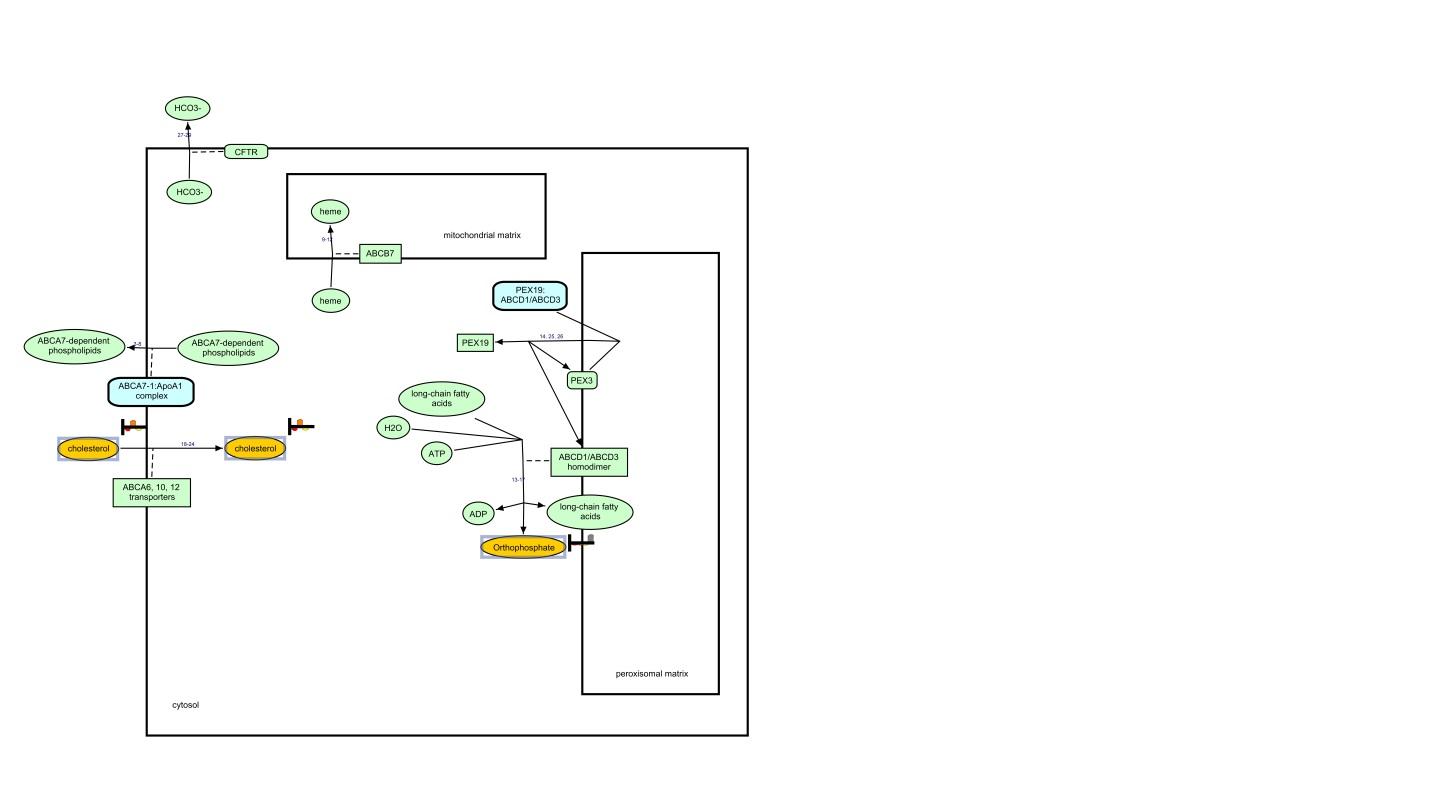


**Fig. S7**. ABC family protein mediated transport, alter metabolites are shown with yellow highlighted color between controls and lung cancer using 32 statistically differentiae metabolites.


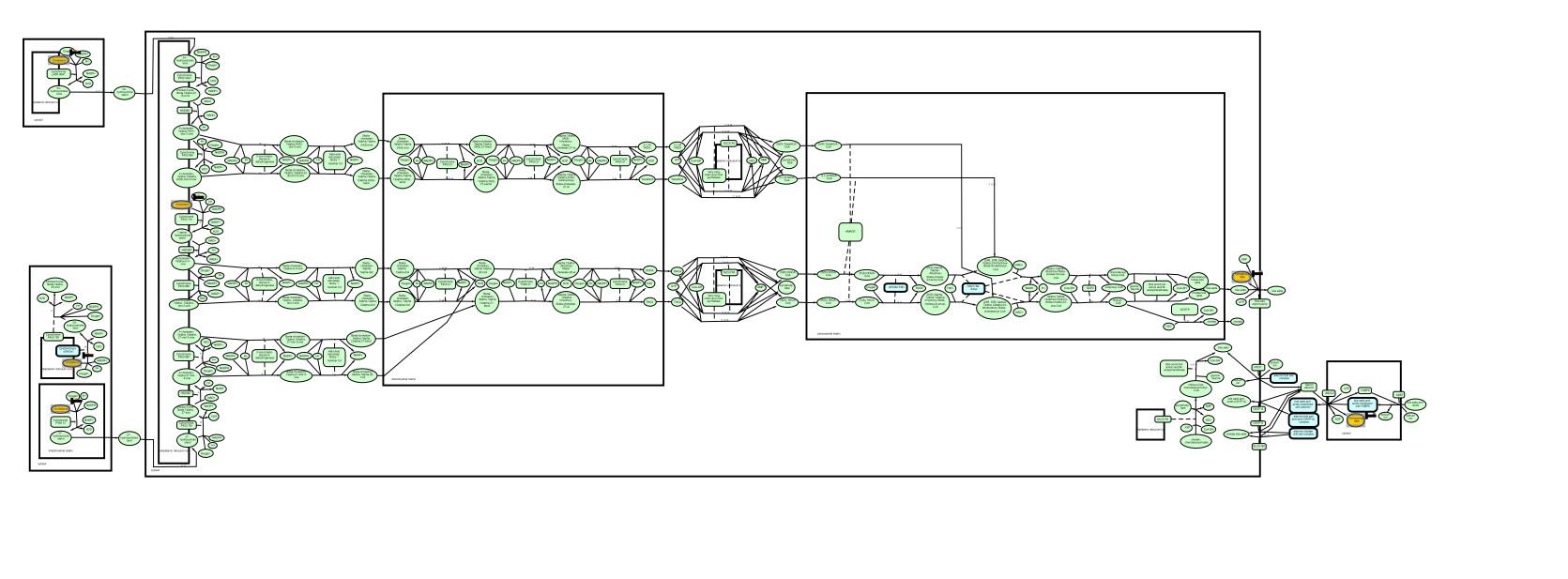


**Fig. S8**. Bile Acid and bile salt metabolism, alter metabolites are shown with yellow highlighted color between controls and lung cancer using 32 statistically differentiae metabolites.


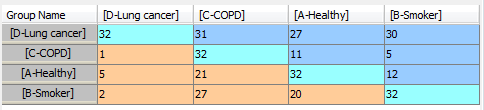


**Table S1.**  Summary of Tukey HSD post hoc. Entities or metabolite found to be differently expressed are represented in blue boxes and significantly expressed, while entities found not to be differently expressed are represented in orange boxes.

| **COPD Vs LC** | **S Vs LC** | **NS Vs LC** | **NS Vs COPD** | **NS Vs S** | **S Vs COPD** |
| --- | --- | --- | --- | --- | --- |
| Octadecanoic acid | Octadecanoic acid | Octadecanoic acid | Octadecanoic acid | Octadecanoic acid | 1-Propene |
| Lactic acid | Lactic acid | Lactic acid |  | Naphthalene |  |
| Phosphoric acid | Phosphoric acid | Phosphoric acid | 1-Propene |  |  |
| Benzoic acid | Benzoic acid | Benzoic acid | Naphthalene |  |  |
| Naphthalene | Naphthalene | Naphthalene |  |  |  |
| d-Glucose | d-Glucose | d-Glucose |  |  |  |
| Altrose | Altrose | Altrose |  |  |  |
| Palmitic acid | Palmitic acid | Cholesterol |  |  |  |
| Octadecanoic acid trimethylsilyl ester | Octadecanoic acid trimethylsilyl ester | Octadecanoic acid trimethylsilyl ester |  |  |  |
| Stearic acid | Stearic acid | Stearic acid |  |  |  |
| 1-Propene | 1-Propene | 1-Propene |  |  |  |
| Cholesterol | Cholesterol | Cholesterol |  |  |  |
| 6.466 | 6.466 | 6.466 | 15.138 | 15.036 | 15.036 |
| 6.627 | 6.627 | 6.627 | 20.794 | 20.794 | 15.138 |
| 7.211 | 7.211 | 7.211 | 21.747 | 23.255 | 23.255 |
| 9.245 | 9.245 | 9.245 | 21.799 | 23.364 |  |
| 9.430 | 9.430 | 9.430 | 23.396 | 23.396 |  |
| 10.948 | 10.948 | 10.948 | 23.452 | 23.452 |  |
| 15.036 | 15.956 | 15.036 | 23.560 |  |  |
| 15.138 | 20.794 | 15.138 | 21.747 |  |  |
| 15.956 | 21.747 | 15.956 | 21.799 |  |  |
| 20.794 | 21.799 | 20.794 |  |  |  |
| 21.747 | 23.255 | 23.364 |  |  |  |
| 21.799 | 23.364 | 23.396 |  |  |  |
| 23.364 | 23.396 | 23.560 |  |  |  |
| 23.396 | 23.452 | 24.409 |  |  |  |
| 23.452 | 23.560 | 25.822 |  |  |  |
| 23.560 | 24.409 | 26.856 |  |  |  |
| 24.409 | 25.822 |  |  |  |  |
| 25.822 | 26.856 |  |  |  |  |
| 26.856 |  |  |  |  |  |

**Table S2.** List of metabolites (32 entities) that are statistically differentially expressed between three controls, healthy Non-Smokers (NS), Smokers (S), Chronic Obstructive Pulmonary Disease (COPD) and Lung Cancer (LC) by applying Tukey HSD post hoc. Identified metabolites are shown with their name and unidentified metabolites with their retention time.

|  | Predicted Healthy | Predicted Smoker | Predicted COPD | Predicted Lung cancer | Accuracy |
| --- | --- | --- | --- | --- | --- |
| True Healthy | 49 | 0 | 0 | 5 | 90.741 |
| True Smoker | 3 | 58 | 5 | 0 | 87.879 |
| True COPD | 1 | 1 | 73 | 0 | 97.333 |
| True Lung cancer | 2 | 0 | 0 | 50 | 96.154 |
| Overall Accuracy |  |  |  |  | 93.117 |

**Table 3.** Summary report of PLS-DA Models generated on MPP from healthy volunteers (n = 54), smokers (n = 66), COPD (n = 75) and lung cancer patients (n = 52).
